# Self-Reported Training Needs and Continuing-Education Preferences of the School Medicine Workforce in Romania: A National Cross-Sectional Survey

**DOI:** 10.3390/healthcare14193245

**Published:** 2026-10-01

**Authors:** Bogdan Florin Covaliu, Daniela Rajka, Cosmin Cristescu, Petru Sandu, Ioana Roxana Bordea, Alina-Ioana Forray

**Affiliations:** 1 Department of Community Medicine, “Iuliu Hațieganu” University of Medicine and Pharmacy, 400012 Cluj-Napoca, Romania; 2 The Society of Doctors from Children’s and Youth Collectivities, 400427 Cluj-Napoca, Romania; 3 National Institute of Public Health—Regional Public Health Centre Cluj, 400349 Cluj-Napoca, Romania; 4 Department of Oral Rehabilitation, Faculty of Dentistry, “Iuliu Hațieganu” University of Medicine and Pharmacy, 400012 Cluj-Napoca, Romania

**Keywords:** school health services, school nursing, health workforce, needs assessment, capacity building, continuing medical education, mental health, cross-sectional studies, Romania, adolescent health

## Abstract

**Highlights:**

**What are the main findings?**

- Among 683 school medical personnel surveyed across all eight development regions, the largest assessment of this workforce in Romania to date, 99.0% expressed interest in further continuing professional development (CPD); among generalist physicians and nurses, the highest self-reported training needs were for emotional and school-adjustment disorders (59%), major emergency first aid (58%), and principles of healthy nutrition (56%), each rated a high or very high need.
- Training needs differed significantly across professional roles in digital skills, ethics, and social and child rights and between generalist physicians and nurses in the clinical and health-education domains (Kruskal–Wallis *p* ≤ 0.033; all effect sizes small, ε^2^ ≤ 0.023); respondents most often preferred a hybrid (in-person plus online; 50.2%) and simulation-led (63.4%) learning model, with insufficient time (39.8%) and cost (37.5%) the most common barriers.

**What are the implications of the main findings?**

- These exploratory, self-reported findings inform the design of a national CPD programme in Romanian school medicine: a modular, hybrid, simulation-based curriculum prioritising mental health, major emergency first aid, nutrition, and staff–family communication, offered free of charge to address the reported cost barrier and scheduled around the workforce’s time constraints.
- Aligned with the WHO 2021 global school-health-services package, this study, the largest assessment of the Romanian school-medicine workforce’s CPD needs to date, directly informs the MED4SCHOOL national programme.

**Abstract:**

Background/Objectives: The continuing professional development (CPD) needs of school medical personnel in Romania have not been characterised at a national scale. We quantified the workforce’s self-reported training needs to inform a national CPD programme. Methods: We conducted a cross-sectional, self-administered online survey (July–September 2025) of school physicians, nurses, dental physicians, and dental nurses, recruited by open invitation through the national school-medicine association’s channels. Training needs (59 role-specific items in seven thematic domains) were rated on a 5-point Likert scale; internal consistency, cross-role contrasts, and regression predictors of the overall training-need score were assessed. Results: The sample comprised 683 respondents (97.4% female; all eight development regions). Interest in further training was near-universal (99.0%); respondents preferred blended delivery (50.2%) and practical simulations (63.4%), with time (39.8%) and cost (37.5%) the leading barriers. Among generalist physicians and nurses, the highest-rated needs were emotional and school-adjustment disorders (59% high or very high need), major emergency first aid (58%), and principles of healthy nutrition (56%); across all roles, communicating with parents ranked highest (53%). Training needs differed by profession for digital skills, ethics, and social and child rights and between generalist professions for the clinical and health-education domains (*p* ≤ 0.033; all effect sizes small); internal consistency was high (α = 0.87–0.96). Conclusions: This national-scale needs assessment, the largest to date for this workforce, identifies self-reported priorities in mental health, emergencies, nutrition, and communication and a strong preference for blended, applied delivery. These exploratory findings inform the MED4SCHOOL national programme, including free-of-charge delivery, without establishing its causal effect on participation; transferability depends on local scope of practice and financing.

## 1. Introduction

School health services (SHSs) reach children and adolescents where they spend most of their day and provide a scalable platform for promotive, preventive, and curative care, including for children who are otherwise outside routine health-system contact [1,2,3]. In 2021, the World Health Organization (WHO), in partnership with UNESCO, issued its first global guideline on school health services, which strongly recommends comprehensive SHSs in all countries and provides an evidence-based package of interventions spanning health promotion, health education, screening, immunisation and preventive interventions, clinical assessment and care, and service-management functions [1,2,4]. The accompanying WHO global School Health Services Survey, completed by 439 experts from 81 countries, categorised the suitability of each candidate intervention for inclusion in routine SHS, generating an international benchmark for national systems [5,6]. Systematic review evidence further indicates that school-based interventions for anxiety, depression, obesity, dental caries, visual disorders, asthma, and adolescent sexual and reproductive health are among the most cost-effective public-health strategies for reaching young populations [3,7,8,9]. At least 102 countries provide some form of SHS, yet the workforce staffing these services is unevenly characterised in national health systems’ planning [1,3,10].

Across Europe, the organisation, governance, scope of practice, and workforce composition of SHSs vary markedly: a WHO Regional Office for Europe typology distinguishes five organisational models, defined by whether dedicated school-health staff are employed and whether care is delivered on school premises or in the community [11], and seven-country and 30-country mappings show that nearly all European countries operate SHSs, with formal services absent in a small minority of states (notably Spain and the Czech Republic) [12,13]. Across the 30 EU and EEA countries surveyed by the Models of Child Health Appraised (MOCHA) consortium, school nurses are the most widely deployed professionals, followed by physicians and psychologists; in 11 of the 30 countries, SHS staff work in multiprofessional teams that may also include social workers and dentists [13]. Workforce expenditure is small, below 0.7% of total health expenditure in comparable five-country data [14]. In 20 of the 30 countries surveyed by MOCHA, specific school-medicine training is not required for entry into the workforce, and continuing professional development (CPD) infrastructure is heterogeneous and often absent [13]. School-based mental-health provision varies particularly widely in the professions involved and in coverage [15,16], and reviews of Central- and Eastern-European (CEE) systems have flagged persistent underinvestment in school mental-health services, weak protocols, unclear roles, and few standardised tools [17,18].

Recent evidence on the disease burden of school-aged populations strengthens the case for comprehensive SHSs. Meta-analyses indicate that clinically elevated depressive and anxiety symptoms affected roughly one in four and one in five young people globally during the COVID-19 pandemic, approximately double pre-pandemic estimates [19]. European meta-analytic data show depression rates 1.36 times higher during the pandemic, with larger effects where school closures were more stringent [20]. Longitudinal cohort data show a corresponding rise in overall mental-health problems from 18% to 28–31% [21], and global modelling estimated 27.6% and 25.6% increases in major depressive and anxiety disorder prevalence in 2020, with larger effects in younger age groups and females [22].

In Romania, the national survey on child well-being in school found high depression risk more common in girls than boys (6.8% vs. 3.8%) [23]; the HBSC Romania study documents a comparable adolescent mental-health and well-being burden [24]; and cross-sectional evidence from Cluj-Napoca youth reports an eating-disorder risk prevalence of 26.5%, with females having 1.6-fold higher odds than males [25]. Childhood overweight and obesity remain elevated across Europe: the WHO European Childhood Obesity Surveillance Initiative (COSI) reports that more than a quarter of 6–9-year-olds across 36 European countries are overweight or obese [26]; forecasts project further increases through 2050 [27,28]. In Romania, the most recent WHO COSI round (2022–2024) found that roughly 28% of 7–9-year-olds were living with overweight or obesity and 12% with obesity [29,30], and over 60% of surveyed youth in Cluj-Napoca reported frequent consumption of ultra-processed foods [25].

Adolescent substance use remains common despite declines in some indicators: European school-survey data document substantial smoking, drinking, and cannabis use in 15–16-year-olds, with cannabis use rising in southern and Balkan countries [31] and latent-class analyses across 25 European countries identify a polysubstance-using group of about 5% of 12–16-year-olds [32]. In Romania, the national Youth Risk Behaviour Survey (YRBSS) recorded lifetime cannabis use of 7.6% in boys versus 4.7% in girls, alongside substantial adolescent tobacco use [33], consistent with HBSC Romania estimates [24]. Schools are also common settings for paediatric injuries that require first-aid response, with traumatic dental injuries frequent in school-aged children, often during school activities [34,35]. In Romania, post-pandemic paediatric oral health has deteriorated, with a four-fold rise in affected permanent teeth among 6-year-old rural children despite a tripling of recommended toothbrushing [36]. Together, these findings indicate heightened, complex, and largely unmet child and adolescent health needs facing the Romanian school medicine workforce [37].

Between September 2021 and August 2023, Romanian school medicine was regulated by the Joint Ministry of Health (MoH) and the Ministry of Education (MoE) Order No. 438/4.629/2021, which defined the organisation of medical and dental care in pre-university and university education and the activities expected of each professional role [38]. That order was replaced by the Methodology of 27 July 2023, approved by Joint MoH and MoE Order No. 2.508/4.493/2023 and in force from August 2023 to January 2026, which extended statutory coverage to ante-preschool children in nurseries, redefined the scope of practice for school physicians, school nurses, school dental physicians, and school dental nurses across all levels of education from nurseries to universities, and listed participation in continuing medical education among the statutory attributions of all four roles [39,40]. Since 11 January 2026, school medicine has been governed by a successor Methodology approved by Joint Order No. 1.503/6.821/2025 [41,42].

According to the most recent national mapping, Romania operates 3348 school and university medical offices, of which 2878 are general and 470 dental practices; 3260 are located in urban areas and only 88 in rural settings; staffing comprises 551 school doctors and 2549 school nurses for approximately 2.84 million pupils, equivalent to one school doctor per 5156 pupils and one school nurse per 1115 pupils, with substantial intercounty variability [43]. Governance is fragmented across the MoH, the MoE, county and local councils (which employ school medical staff under Law 174/2011), and the county Public Health Directorates, creating coordination and accountability gaps [44]. National-level reporting is partial: in 2017, balance-examination results were submitted for only 25% of the 2.2 million children aged 3–14, reflecting incomplete territorial coverage and weak data systems [43]. Roșioară and colleagues note that Romanian spending on prevention is the second lowest in the European Union, primary-care spending is the lowest in absolute terms, school medical offices “play a vital role in detecting treatable conditions” but require stronger intersectoral collaboration and equipment, and there are no national oral-health prevention programmes nor a national caries epidemiological survey [37]. These structural gaps weigh most on rural children, children from Roma communities, and children with neurodevelopmental and special-educational needs, whose access to age-appropriate care relies disproportionately on the SHS platform [43,44,45,46].

The Romanian school medicine workforce is also shaped by a wider health workforce crisis: the World Health Organization estimated a global shortfall of 15 million health workers in 2020, projected to ease to approximately 10 million by 2030, with the most acute gaps in the African and Eastern Mediterranean regions [47,48]. The European region, although less affected, faces population ageing, professional out-migration, and uneven geographic distribution, with persistent retention difficulties in Eastern Europe; Romania has historically combined high out-migration of physicians and nurses, maldistribution between urban academic centres and rural areas, and an ageing professional cohort [49,50].

Recent national policy initiatives, including the 2025 Romanian guide for health-workforce attraction and retention developed with WHO technical assistance [51], and a 2025 multistakeholder consensus on health-system resilience priorities [52], stress that workforce capacity-building must be integrated with retention, distribution, and digitalisation policies if national-level impact is to be sustained. A UNICEF Romania case study on primary-healthcare workforce development highlighted understaffing, maldistribution, and gaps in specialised training in school medicine as systemic obstacles to delivering equitable child and adolescent health services [45].

Despite the WHO global call for comprehensive SHSs [1,2] and the European mapping of SHS organisation [13], the CPD needs of the Romanian school-medicine workforce remain insufficiently characterised: a national assessment examined the training needs of school nurses [53], but no study has characterised needs across all four roles at a comparable national scale. The 2023 Methodology expanded the clinical, public-health, and health-promotion competencies expected of the workforce relative to the 2021 order [38,40], and digital health and AI literacy competencies are increasingly required of all primary-care professionals [54,55], without a concurrent national-scale assessment of the workforce’s self-reported training needs. Internationally, structured training-needs assessments, including the Hennessy–Hicks Training Needs Analysis Questionnaire and adapted public-health workforce frameworks, have been used to direct CPD investment toward highest-impact domains [56,57,58,59], and a school-nurse-specific competency framework has identified six core CPD priority areas spanning patient-centred care, communication and collaboration, evidence-based critical thinking, programme implementation, legal and ethical practice, and health education [60]. The WHO global SHS Survey methodology provides a model of expert-based prioritisation adaptable to a national needs assessment [5,6]. The WHO European Region’s 2030 progress review calls for redesigned, adequately financed health systems with children’s health embedded in human-rights commitments, noting that COVID-19 and the war in Ukraine will likely reverse gains [61].

Study aim. This study aimed to characterise, at a national scale, the self-reported training needs and CPD priorities of the Romanian school medicine workforce. Specifically, we sought to (i) describe current participation in CPD and the formats and topics accessed; (ii) quantify perceived training needs across the clinical, public-health, health-education, communication, ethics, social and child-rights, and digital-skills domains; (iii) identify preferred training modalities and delivery formats; and (iv) characterise barriers to participation in CPD.

## 2. Materials and Methods

### 2.1. Study Design

We conducted a cross-sectional, confidential, self-administered online survey of the Romanian school medicine workforce. The study used a needs-assessment design to inform the prioritisation and design of a national capacity-building programme [57,58,59]. The manuscript is written in accordance with the Strengthening the Reporting of Observational Studies in Epidemiology (STROBE) statement for cross-sectional studies [62].

### 2.2. Setting and Population

During the study period, Romanian school medicine was jointly regulated by the MoH and the MoE through the Methodology approved by Joint Order No. 2.508/4.493/2023, which replaced Joint Order No. 438/4.629/2021 in August 2023 and defines the organisation of medical and dental care in pre-university and university educational establishments. School medicine units are deployed in nurseries, schools, high schools, and universities across all 41 counties and Bucharest, staffed by school physicians, school dental physicians, school nurses, and school dental nurses [38,39,40,41,42]. Recruitment and data collection were conducted between 24 July and 30 September 2025.

The sampling frame consisted of the professional community of Romanian school medicine practitioners reachable through the Society of Doctors from Children’s and Youth Collectivities (SDCYC), the only nationally recognised professional association for school medicine in Romania, with more than 200 dues-paying members across all 41 counties and Bucharest. SDCYC is accredited as a Continuing Medical Education provider by the Romanian College of Physicians (since 2010) and the Romanian College of Dentists (since 2021). The questionnaire was distributed through the association’s three established communication channels: (i) the association website, www.medicinascolara.eu (accessed on 24 September 2026); (ii) the Romanian *Journal of School and University Medicine*; and (iii) the SDCYC national congress mailing list. We adopted a non-probability, open-invitation sampling strategy: every professional reached through these channels was invited to participate. No paper version was administered, and no targeted reminders were sent to individual members.

Eligible respondents were medical personnel who (i) were employed in a school-medicine unit in Romania at the time of survey completion, (ii) were able to complete a self-administered online questionnaire in Romanian, and (iii) provided active electronic informed consent. No additional inclusion/exclusion criteria were applied.

### 2.3. Variables

The primary outcome was the self-rated need for training across 59 items (17 administered to all professional roles, 24 to generalist physicians and nurses only, and 18 to dental personnel only), grouped into seven thematic domains and measured on a 5-point Likert scale (1 = “very low need” to 5 = “very high need”). Two secondary outcomes were derived from the primary outcome: (i) a binary “high-need” indicator (1 if Likert ≥ 4, 0 otherwise) at the item and domain level, and (ii) a continuous domain mean training-need score (average of items within domain). An overall mean training-need score was computed as the average of the five domain means administered to all professional roles (communication; social and child rights; ethics; digital skills; and the common public-health items; 17 items in total) and served as the dependent variable for multivariable regression.

Predictors and potential confounders assessed were profession (school physician, school dental physician, school nurse, school dental nurse), sex, age (categorised), years of experience in school medicine (categorised), residence type (urban/rural), and prior participation in school-medicine training in the preceding 2 years. Potential effect modifiers were profession and residence type, examined through stratified descriptive analyses and the multivariable model.

### 2.4. Data Sources and Measurement

A custom self-administered electronic questionnaire was developed for this study because no validated Romanian-language instrument exists that simultaneously (i) reflects the scope of practice legally assigned to school-medicine personnel in Romania and (ii) maps onto the WHO global package of school health services. The instrument was therefore designed from first principles, anchored to two independent reference frames: the Romanian regulatory framework in force during instrument development and data collection, namely the Methodology approved by Joint Order No. 2.508/4.493/2023, which defines the functional attributions of school medicine personnel [39,40], and the WHO global School Health Services (SHS) intervention menu [4].

The Methodology approved by Joint Order No. 2.508/4.493/2023 enumerates the activities that each professional role is expected to perform, including clinical examination of pupils and students; verification of vaccination status and immunisation in special epidemiological situations; hygiene and food-safety inspection of educational establishments and of the teaching–learning process; school health education and risk-behaviour prevention; epidemiological triage, outbreak investigation, and intervention; dispensary follow-up of children with chronic conditions; dental prophylaxis and treatment of oro-dental lesions in school dental offices; ethical and confidentiality obligations toward minor patients; and administrative reporting in national health information systems [39,40]. The training-need section of the questionnaire was constructed to operationalise these legally mandated activities as discrete training topics, thereby ensuring scope-of-practice coverage by design rather than by post hoc rationalisation.

The WHO, building on the global School Health Services package, recommends an integrated set of evidence-based interventions delivered to children and adolescents through school health services, spanning health protection, health promotion, and clinical care functions [1,2,5]. Each candidate item in our instrument was cross-mapped to one or more of the WHO SHS interventions and to the corresponding attribution in the annexes of the 2023 Methodology; interventions delivered exclusively outside the Romanian school-medicine scope of practice (for example, those carried out by hospital paediatric services or by community general practitioners) were not retained.

The questionnaire was developed iteratively by the multidisciplinary study team, whose combined expertise spans medical education, community medicine and public health (B.F.C., C.C., A.I.F., P.S.), school-medicine practice and national-level professional governance (D.R.), and dental medicine (I.R.B.). Activities legally attributed to school-medicine personnel were extracted from Annexes 1–4 of the Methodology approved by Joint Order No. 2.508/4.493/2023 and cross-mapped to the interventions of the WHO School Health Services Survey (2019); items relevant only outside the Romanian scope of practice were excluded, while items anchored in the Romanian legal mandate but absent from the WHO menu were retained as Romania-specific scope items. Two emerging competency areas not addressed by either source, generic digital skills and the use of large language models in clinical practice, were added inductively after team discussion, because both bear directly on the day-to-day capacity of school-medicine personnel in 2025.

The resulting items were grouped into seven thematic domains (clinical care; hygiene and public health; health education and sexual education; social domain and children’s rights; ethics; digital skills and innovation; and communication), rephrased from administrative legal language into respondent-friendly Romanian in lay vocabulary, and reviewed by the full author team to verify scope-of-practice coverage, item clarity, and the avoidance of double-barrelled wording. The questionnaire was developed natively in Romanian, drawing on the wording of the national regulatory texts, with simplification into lay vocabulary guided by health-literacy principles. Prior to launch, the instrument was pre-tested internally by the author team to establish face validity, verify skip-logic functionality, estimate completion time, and refine item wording; no external pilot testing or cognitive interviewing was conducted. Role-specific skip logic was implemented in Google Forms (Google LLC, Mountain View, CA, USA) to route each respondent, on the basis of their stated profession, to one of four item blocks. The item content of the health-education domain differed between the generalist and dental blocks (the dental version covered healthy lifestyle, oral hygiene, dietary hygiene, and the prophylaxis of dental caries and dento-maxillary anomalies); these items were therefore analysed as role-specific. One public-health item (active surveillance of children with chronic conditions) used role-adapted wording in the dental blocks and is footnoted accordingly in the tables.

The final questionnaire was organised in four parts, in the order administered: (i) sociodemographic characteristics (age band, sex, county, urban/rural domicile); (ii) professional activity, comprising workplace characteristics, years of practice, type and size of school medicine units served, recent participation in continuing professional development (event types, topic areas, mode of access (free of charge versus fee-based)), interest in further training, an open-text item on barriers to participation, and preferences regarding training format (in-person, online, hybrid), teaching methods, and host university centres; (iii) professional category, which routed each respondent to the corresponding role-specific block; and (iv) role-specific training-need blocks covering the seven thematic domains, scored on the 5-point Likert scale described above, each concluding with an open-text item inviting additional training topics not covered by the closed-ended items. The seven thematic domains were: clinical care; hygiene and public health; health education and sexual education; social domain and children’s rights; ethics; digital skills and innovation; and communication. Items were administered to all professional roles or, where role-specific, routed via Google Forms skip logic based on the respondent’s stated profession (school physician, school nurse, school dental physician, or school dental nurse). The full English-translated instrument, with item ordering, response scales, and skip logic matching the deployed Romanian version, is reproduced in Supplementary Material S1. The English version was produced by two bilingual researchers from the author team (A.I.F. and C.C.) following the WHO process for translating and adapting instruments, using forward translation followed by consensus back-translation [63]. The English version was then checked against the deployed Romanian instrument item by item, across all 51 form elements, covering item wording, response options, scale anchors, grid structures, and skip logic; no discrepancy affecting item meaning was identified, and minor wording differences were harmonised. All variables were measured by the same instrument administered identically to every respondent; no intermethod comparability adjustments were required.

### 2.5. Study Size

No a priori power calculation was performed because the study was designed as an exploratory, open-invitation needs assessment rather than a hypothesis test. All professionals reachable through SDCYC channels were invited; the achieved analytic sample of *N* = 683 consenting respondents reflects this open-invitation strategy. Because the SDCYC website and journal are publicly accessible and the congress mailing list extends beyond dues-paying members, a denominator-based response rate cannot be computed. The 644 generalist respondents correspond to approximately one-fifth of the 3100 school physicians and nurses enumerated in the most recent national mapping (551 school physicians and 2549 school nurses) [43]; because the number of eligible professionals reached through the recruitment channels cannot be determined, this proportion should be interpreted as an indicator of workforce coverage rather than as a response rate. Accordingly, “national-scale” is used throughout this article to denote the countrywide reach of the recruitment channels and the geographic spread of respondents across all eight development regions, not the representativeness of the sample for the Romanian school-medicine workforce.

### 2.6. Operationalisation and Coding of Variables

Training-need ratings were collected on a 5-point Likert scale for each of the 59 individual items (1 = very low need, 2 = low need, 3 = moderate need, 4 = high need, 5 = very high need). Item ratings were analysed both as ordinal variables (medians, interquartile ranges, Kruskal–Wallis and Mann–Whitney tests) and, for descriptive priority ranking, dichotomised at ≥4 to yield an item-level “high or very high need” indicator, following the conventional top-two-box approach for 5-point scales. Domain-level scores were constructed as the arithmetic mean of constituent items within each of the seven thematic domains (plus four dental-specific subdomains for the dental subgroup), computed for respondents answering every constituent item (complete-case), and were treated as approximately interval for the purpose of domain-level descriptive statistics, internal-consistency analyses (Cronbach’s α with 95% bootstrap confidence intervals, McDonald’s ω), and the multivariable linear regression of overall mean training-need score.

Age was retained as a categorical variable using the nine bands collected in the questionnaire (25–29, 30–34, 35–39, 40–44, 45–49, 50–54, 55–59, 60–65, and over 65 years); for the multivariable regression it was additionally coded as an ordinal score from 1 (25–29 years) to 9 (over 65 years), with a one-step increase corresponding to a five-year increment in the lower eight bands. Years of experience in school medicine was collected as a free-text numeric value and categorised during analysis into eight five-year bands (0–4, 5–9, 10–14, 15–19, 20–24, 25–29, 30–34, and 35 or more years); it was coded for the regression as an ordinal score from 0 (0–4 years) to 7 (35 or more years), again with a one-step increase corresponding to a five-year increment in the lower seven bands.

Multiselect items (educational establishments served, training-event types attended, course topics attended, and preferred training formats and learning methods) were retained as binary indicators per response option (1 = endorsed, 0 = not endorsed); column percentages for these items were computed over the full consenting analytic sample (*N* = 683) and do not sum to 100% within a variable. For each course topic, attendance and access mode were recorded in the questionnaire as two tick-all-that-apply indicators per topic (free of charge; fee-based); a four-category variable (did not attend, free of charge only, paid out of pocket only, mixed) was derived from these indicators during analysis, and the two binary indicators used in the per-topic McNemar comparison were defined from it (any free-of-charge attendance, taking the value 1 if the four-category variable was coded “free of charge only” or “mixed”; any paid-out-of-pocket attendance, taking the value 1 if the variable was coded “paid out of pocket only” or “mixed”).

Continuous and ordinal descriptive statistics (mean, standard deviation, 95% confidence interval, median, interquartile range) were reported alongside categorical summaries (counts and column percentages) as appropriate to each variable. The training-need items were configured as mandatory in the deployed form (Supplementary Material S1), and the raw export contained no item-level missing data on them; the only missing values in the analytic dataset are three free-text answers on years of experience that could not be converted to a number of years (Section 3.1). Barriers to participation were collected as a single open-text item and coded into barrier categories by directed content analysis, as described in Section 2.7; the resulting category indicators were treated as binary variables per respondent.

### 2.7. Statistical Methods

Quantitative analyses were performed in IBM SPSS Statistics (version 31.0 for macOS; IBM Corp., Armonk, NY, USA). All statistical tests were two-tailed with significance set a priori at α = 0.05. Descriptive statistics included counts and percentages for categorical variables, and means with standard deviations, 95% confidence intervals, medians, and interquartile ranges for continuous and Likert-scale variables. For the multi-item training-need domains, internal consistency was assessed using Cronbach’s α (with 95% bootstrap confidence intervals based on 1000 resamples) and McDonald’s ω, along with the mean inter-item correlation [64,65]. The dimensionality of the 17 items administered to all professional roles was examined by exploratory factor analysis (principal axis factoring with direct oblimin rotation), with the number of factors to retain guided by Horn’s parallel analysis; a five-factor solution corresponding to the a priori domains was also extracted as an exploratory, theoretically imposed solution. Both solutions are reported in Supplementary Material S2; neither constitutes a psychometric validation of the instrument. Inferential comparisons used the χ^2^ test (or Fisher’s exact test where expected cell counts were < 5) for categorical contrasts, the Kruskal–Wallis test (with ε^2^ as the effect-size measure) for ordinal contrasts across three or more groups, and the Mann–Whitney U test (with rank-biserial r as the effect size) for two-group ordinal contrasts. Within-respondent contrasts of free-of-charge versus paid-out-of-pocket attendance for each of the eight course topics used McNemar’s test (continuity-corrected χ^2^, confirmed by the exact binomial test), with Bonferroni correction across the eight contrasts (corrected α = 0.00625). Effect-size magnitudes were interpreted using Cohen’s conventional thresholds [66,67,68]. Confounder adjustment was implemented through a multivariable linear regression with the overall mean training-need score as the outcome and profession, sex, age category, years of experience, domicile environment, and prior training as covariates. Model assumptions were assessed graphically (residual-versus-fitted and Q–Q plots) and numerically through variance inflation factors (VIF < 5 indicating acceptable multicollinearity). Conventional standard errors are reported; in a sensitivity analysis using heteroscedasticity-consistent (HC3) standard errors, the joint Wald test (*p* = 0.118) and the coefficient-level inferences, including the exploratory dental-physician contrast (*p* = 0.299), agreed with the conventional model. Adjusted R^2^ and unstandardised regression coefficients (B) with 95% confidence intervals are reported.

Pre-specified subgroup analyses were conducted by profession, sex, urban/rural domicile, and prior training. A sensitivity analysis re-ran the primary descriptive and inferential comparisons excluding the dental subgroup (*n* = 39) to assess robustness to the smaller dental stratum and to verify that overall conclusions were not driven by role-specific patterns.

Responses to the two open-ended items were analysed by directed content analysis against written codebooks [69]. For the barriers item, a nine-category coding framework was developed deductively from the barrier examples embedded in the questionnaire prompt (course costs, lack of time, topics not suited to needs, lack of locally organised events) and refined during a first reading of the responses, which added categories for information, organisational support, delivery format, personal circumstances, and the absence of barriers; two initially separate availability categories were merged after a reliability assessment. Because respondents frequently named several barriers, each response could be assigned to between one and four categories: 633 responses (92.7%) named at least one barrier, 36 (5.3%) stated or implied that the respondent faced none, and 14 (2.0%) contained no substantive answer (a placeholder character entered to pass the compulsory item, “do not know”, or an off-topic remark); the last two groups are tabulated separately.

For the additional-training-topics item, initial open codes were consolidated into 13 mutually exclusive theme groups, and each response was assigned once to its dominant theme; 195 responses (28.6%) contained a valid training-topic suggestion. Category definitions and example statements for both codebooks are provided in Supplementary Material S3. All responses to both items were coded by one author (A.I.F.) and independently recoded in full, blind to the first coding, by a second co-author (C.C.). Agreement between the two codings was 91.4% at the theme-group level for the topics item (Cohen’s κ = 0.80). For the barriers item, reliability was assessed per category: Cohen’s κ averaged 0.81 across the nine categories (range 0.39–0.97, with the lowest values in categories endorsed by fewer than 4% of respondents), and the two codings assigned an identical set of categories to 89.9% of responses. Disagreements were resolved by consensus and adjudicated by A.I.F., B.F.C. and C.C.; the consensus codes provide the reported frequencies for both items.

### 2.8. Ethics

The study was conducted in accordance with the Declaration of Helsinki and approved by the Ethics Commission of the “Iuliu Hațieganu” University of Medicine and Pharmacy Cluj-Napoca (protocol code DEP122/26.03.2025, approved 24 July 2025). The questionnaire opened with a data-protection notice and an explicit consent question; informed consent was obtained electronically from all subjects prior to questionnaire completion, and participants who did not provide consent were not advanced to the survey items. The survey platform recorded respondents’ e-mail addresses. Only the corresponding authors (D.R. and A.I.F.) had access to the file containing them; the addresses were not used in any analysis and were removed from the analytic dataset. Potential duplicate submissions were screened by comparing the recorded e-mail addresses; no duplicate submissions were identified. Personal data processing complied with Regulation (EU) 2016/679 (GDPR) and Romanian Law 190/2018; the analytic dataset contains no directly or indirectly identifying information and cannot be linked back to individual respondents.

## 3. Results

### 3.1. Respondent Profile

Of the 703 survey records initially received, 683 respondents (97.2%) provided active consent and constitute the analytic sample (*N* = 683). Key sociodemographic and professional characteristics are summarised in Table 1.

The sample was predominantly female (97.4%) and resident in urban localities (85.5%). The workforce was skewed toward mid-career and senior staff: 48.0% of respondents were aged 50 years or older, with the 45–49 and 50–54-year bands jointly modal (26.5% each), followed by 55–59 years (17.4%); only 5.4% were younger than 35 years. Generalist school nurses formed the largest professional group (72.3%), followed by generalist school physicians (22.0%); dental personnel together accounted for 5.7% of the sample (dental physicians, 4.1%; dental nurses, 1.6%).

Most respondents worked in urban county-capital settings (84.9%), with 12.6% in non-county-capital urban localities and 2.5% in rural settings. Years of professional experience showed a broad distribution consistent with the age profile: 24.9% of respondents reported 0–4 years of experience (early-career), 21.3% reported 5–9 years (mid-career entrants), and 53.8% had 10 or more years of experience (senior staff), with 26.6% having accumulated 20 or more years of practice.

All eight development regions of Romania were represented. The North-East (24.7%), North-West (22.7%), and Centre (19.6%) regions accounted for two-thirds of respondents (67.0%); the South-East, South-Muntenia, South-West Oltenia, West, and Bucharest-Ilfov regions together made up the remaining 33.0%.

Respondents typically served more than one type of educational establishment. The most commonly reported settings were kindergartens (52.4%), lower-secondary schools (47.1%), high schools (42.0%), and primary schools (32.5%); smaller proportions reported covering nurseries (17.3%), vocational schools (11.7%), special-needs schools (5.0%), and universities (2.5%).

The distribution of respondents differed significantly across professional roles for sex, age, residence type, development region, years of experience, and every school-unit category except special-needs schools (the nursery contrast at *p* = 0.007, all other significant contrasts at *p* < 0.001); for work environment (*p* = 0.709) and special-needs schools (*p* = 0.156), no significant difference was detected. Generalist physicians were proportionally more likely than nurses to serve in lower-secondary schools (76.0% vs. 37.7%), high schools (71.3% vs. 30.6%), and primary schools (61.3% vs. 23.1%), reflecting the broader catchment typically assigned to physicians in the Romanian school-medicine system.

### 3.2. Continuing-Education Engagement: Participation, Preferences, and Barriers

Self-reported participation in continuing-education activities was high overall and is summarised in Table 2. Courses were the most frequently attended event type, with 88.4% of respondents having participated in at least one (Table 2). The other six event formats recorded lower but non-negligible participation, in descending order of frequency: physical congresses (48.3%), online congresses (44.8%), workshops and webinars (both 34.7%), other events (32.8%), and symposia (32.2%).

Course-topic participation showed a strong skew toward clinically applied and child-focused content (Table 2). The most frequently attended course topics were emergencies and first aid (70.6%) and child pathology (68.5%), followed by health education (51.5%), hygiene and disinfection (40.3%), communication with patients (38.2%), patient rights (30.9%), ethics (23.3%), and digitalisation and innovation (15.8%). A consistent and statistically robust finding across all eight topics was the predominance of free-of-charge access. For each topic, the proportion of respondents who attended free-of-charge sessions was significantly higher than the proportion who paid out of pocket (McNemar’s test, all *p* < 0.001; all contrasts remained significant after Bonferroni correction for eight tests, α-corrected = 0.00625). The free-versus-paid asymmetry was largest in absolute terms for emergencies and first aid (free, 60.0%; paid, 13.3%; χ^2^(1) = 218.4) and smallest, though still highly significant, for digitalisation and innovation (free, 12.0%; paid, 4.0%; χ^2^(1) = 27.3). Across the eight topics, between 1 and 24 respondents per topic reported attending the same topic via both modes (the “mixed” group).

Beyond prior engagement, the workforce reported a high level of motivation for further training. Interest in future continuing-education activities was nearly universal, with 99.0% of respondents (676/683) expressing willingness to participate. Preferences for delivery format converged on a blended model: a mixed in-person plus online format was favoured by 50.2% of respondents, ahead of in-person-only delivery (29.6%) and online-only delivery (20.2%). Among preferred learning methods, practical simulations and case scenarios ranked first (63.4%), followed by online learning and webinars (47.3%) and clinical-case analysis (44.9%). Preferred host university centres (multiselect) were led by Cluj-Napoca (30.0%), Iași (25.6%), and Bucharest (19.8%), followed by Târgu Mureș (13.9%), Timișoara (9.7%), Craiova (7.8%), Constanța (7.3%), and Sibiu (5.0%), with other centres named by 5.7% of respondents.

Barriers to participation in continuing-education activities are summarised in Table 2. The most frequently reported barriers were time and schedule constraints (39.8%), cost (37.5%), and the limited local availability of training events (36.6%), followed by the relevance or adaptation of available content (12.6%). Less commonly reported barriers were organisational or employer issues (3.1%), information or communication gaps (1.0%), the format of training events (1.0%), and specific personal circumstances (0.9%). Thirty-six respondents (5.3%) stated that they faced no barriers, and 14 (2.0%) gave no substantive answer (a placeholder character, “do not know”, or an off-topic remark).

### 3.3. Item-Level Training Needs

Item-level descriptive statistics for all 59 training-need items are reported across three companion tables that follow the survey’s skip-logic structure: Table 3a presents items administered to all professional roles (all HCPs); Table 3b presents items administered only to generalist physicians and nurses (general HCPs); and Table 3c presents items administered only to dental personnel (dental HCPs subgroups). Within each table, items are grouped by thematic domain and ranked by mean rating within the domain.

Across the 17 items administered to all professional roles (Table 3a), the top-rated items by combined “high” plus “very high” need were Communicating with parents (mean 3.58, SD 1.21; 52.7%), Communicating with authorities (3.57, 1.20; 50.8%), Communicating with children (age-appropriate) (3.53, 1.21; 50.8%), Child abuse (physical, neglect, emotional, sexual) (3.52, 1.19; 49.8%), and Motivation and persuasion (3.50, 1.19; 49.2%).

**Figure 1:**
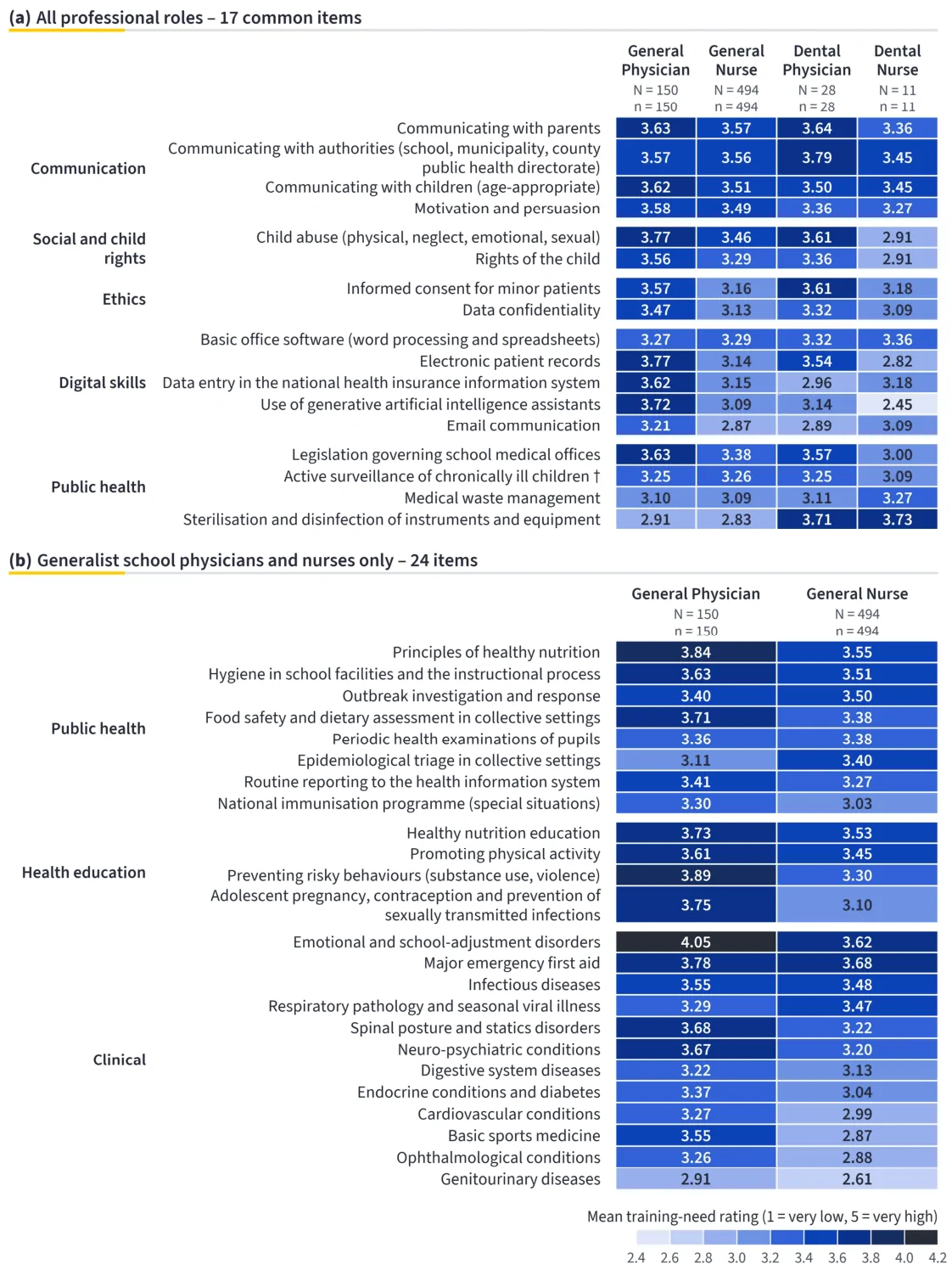
Mean self-reported training-need ratings by professional role (5-point scale: 1 = very low need, 5 = very high need), grouped by thematic domain and presented by survey skip-logic block: (**a**) the 17 items administered to all four professional roles; (**b**) the 24 items administered only to generalist school physicians and school nurses. Each column heading gives the subgroup size (*N*) and the item-level denominator (*n*); the two coincide for every role (150 general physicians, 494 general nurses, 28 dental physicians, 11 dental nurses). Dental estimates are exploratory given the small subgroups. Cell colour is binned in 0.2-point bands spanning the observed range of item means (2.45–4.05), on the same scale as Figure 2. † Worded as active surveillance of children with chronic oro-dental conditions in the dental questionnaire blocks. The 18 items administered only to dental personnel are shown in Figure 2.

Among items administered only to generalist physicians and nurses (Table 3b; Figure 1), the top-rated items were Emotional and school-adjustment disorders (mean 3.72, SD 1.14; 59.3%), Major emergency first aid (3.70, 1.16; 57.6%), Principles of healthy nutrition (3.62, 1.29; 55.9%), Healthy nutrition education (3.58, 1.24; 53.3%), Hygiene in school facilities and the instructional process (3.54, 1.16; 49.4%), Outbreak investigation and response (3.47, 1.23; 49.7%), and Food safety and dietary assessment in collective settings (3.46, 1.28; 50.9%).

**Figure 2:**
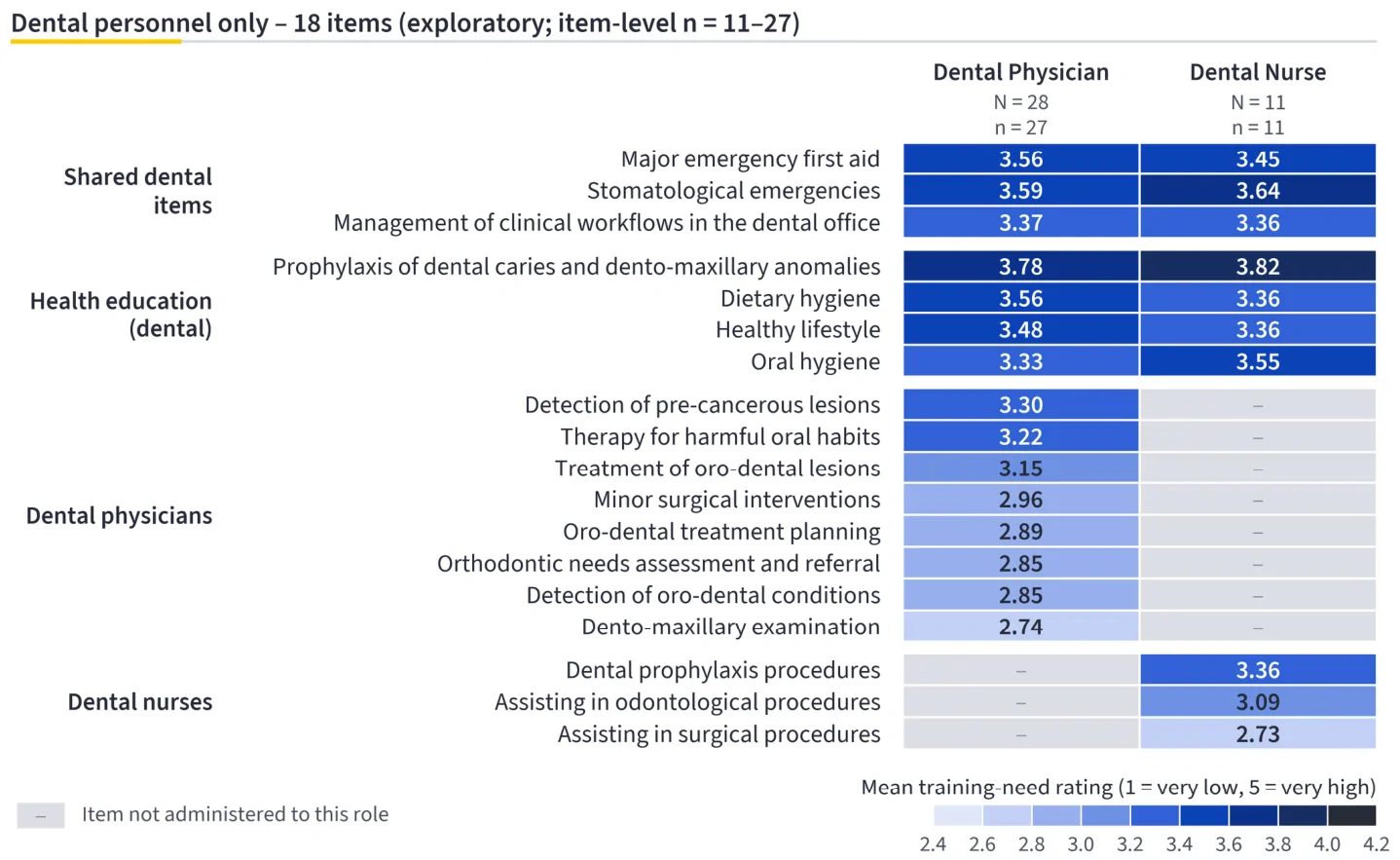
Mean self-reported training-need ratings for the 18 items administered only to dental personnel, by dental role (5-point scale: 1 = very low need, 5 = very high need). All estimates are exploratory given the small subgroups. Each column heading gives the subgroup size (*N*: 28 dental physicians and 11 dental nurses) and the item-level denominator (*n*); one dental physician was routed by the survey’s skip logic to the generalist block, so *n* = 27 in every dental-physician cell and 11 in every dental-nurse cell. Grey cells marked with a dash indicate items not administered to that role. Cell colour is binned in 0.2-point bands spanning the observed range of item means (2.45–4.05), on the same scale as Figure 1. Table 3c reports the seven items administered to both dental roles with the roles pooled (*n* = 38); this figure reports them by role, so the means differ.

Items administered only to dental personnel (Table 3c; shown separately by dental role in Figure 2) were derived from small subgroups (*n* = 11 to 38) and produced correspondingly wide 95% confidence intervals. Within this group, the top-rated items were Prophylaxis of dental caries and dento-maxillary anomalies (mean 3.79, SD 1.02; 60.5%; *n* = 38), Stomatological emergencies (3.61, 1.13; 52.6%; *n* = 38), Major emergency first aid (3.53, 1.11; 52.6%; *n* = 38), Dietary hygiene (3.50, 1.08; 50.0%; *n* = 38), and Healthy lifestyle (3.45, 1.03; 39.5%; *n* = 38).

**Table 3:** (**a**) Item-level training needs administered to all professional roles (All HCPs, target *N* = 683). Items are grouped by thematic domain and ranked by mean rating within the domain. (**b**) Item-level training needs administered only to generalist physicians and nurses (General HCPs, *n* = 644). Items are grouped by thematic domain and ranked by mean within domain. (**c**) Item-level training needs administered only to dental personnel (Dental HCPs, subeligibility *n* = 11 to 38). Items are grouped by subeligibility block and ranked by mean within block. All estimates are exploratory given the small subgroups.

(**a**)				
**Domain/Item**	***N***	**Mean (SD)**	**95% CI**	**% ≥ 4 ^a^**
**Communication**				
Communicating with parents	683	3.58 (1.21)	3.49, 3.67	52.7
Communicating with authorities (school, municipality, county public health directorate)	683	3.57 (1.20)	3.48, 3.66	50.8
Communicating with children (age-appropriate)	683	3.53 (1.21)	3.44, 3.62	50.8
Motivation and persuasion	683	3.50 (1.19)	3.41, 3.59	49.2
**Social and Child Rights**				
Child abuse (physical, neglect, emotional, sexual)	683	3.52 (1.19)	3.43, 3.61	49.8
Rights of the child	683	3.35 (1.14)	3.26, 3.43	42
**Ethics**				
Informed consent for minor patients	683	3.27 (1.28)	3.18, 3.37	41.4
Data confidentiality	683	3.21 (1.31)	3.11, 3.31	39.2
**Digital Skills**				
Electronic patient records	683	3.29 (1.30)	3.19, 3.39	44.4
Basic office software (word processing and spreadsheets)	683	3.29 (1.23)	3.20, 3.38	42.3
Data entry in the national health insurance information system	683	3.25 (1.29)	3.15, 3.34	43.2
Use of generative artificial intelligence assistants	683	3.22 (1.36)	3.12, 3.32	42.3
Email communication	683	2.95 (1.34)	2.85, 3.05	33.8
**Public Health ^b^**				
Legislation governing school medical offices	683	3.44 (1.17)	3.35, 3.53	45.7
Active surveillance of chronically ill children ^†^	683	3.25 (1.24)	3.16, 3.34	41.1
Medical waste management	683	3.09 (1.26)	3.00, 3.19	35.3
Sterilisation and disinfection of instruments and equipment	683	2.90 (1.31)	2.80, 2.99	30.7
(**b**)				
**Domain/Item**	***N***	**Mean (SD)**	**95% CI**	**% ≥ 4 ^a^**
**Health Education**				
Healthy nutrition education	644	3.58 (1.24)	3.48, 3.68	53.3
Promoting physical activity	644	3.49 (1.20)	3.39, 3.58	49.8
Preventing risky behaviours (substance use, violence)	644	3.43 (1.38)	3.33, 3.54	50.8
Adolescent pregnancy, contraception and prevention of sexually transmitted infections	644	3.25 (1.42)	3.14, 3.36	45
**Public Health**				
Principles of healthy nutrition	644	3.62 (1.29)	3.52, 3.72	55.9
Hygiene in school facilities and the instructional process	644	3.54 (1.16)	3.45, 3.63	49.4
Outbreak investigation and response	644	3.47 (1.23)	3.38, 3.57	49.7
Food safety and dietary assessment in collective settings	644	3.45 (1.28)	3.36, 3.55	50.9
Periodic health examinations of pupils	644	3.38 (1.29)	3.28, 3.48	47.7
Epidemiological triage in collective settings	644	3.33 (1.35)	3.23, 3.44	45.7
Routine reporting to the health information system	644	3.30 (1.25)	3.20, 3.40	43.3
National immunisation programme (special situations)	644	3.09 (1.18)	3.00, 3.18	33.5
**Clinical**				
Emotional and school-adjustment disorders	644	3.72 (1.14)	3.63, 3.81	59.3
Major emergency first aid	644	3.70 (1.16)	3.62, 3.79	57.6
Infectious diseases	644	3.49 (1.13)	3.40, 3.58	49.8
Respiratory pathology and seasonal viral illness	644	3.43 (1.16)	3.34, 3.52	47
Spinal posture and statics disorders	644	3.32 (1.19)	3.23, 3.42	43.2
Neuropsychiatric conditions	644	3.31 (1.19)	3.22, 3.41	43.5
Digestive system diseases	644	3.15 (1.06)	3.07, 3.23	34.2
Endocrine conditions and diabetes	644	3.11 (1.17)	3.02, 3.21	36.6
Cardiovascular conditions	644	3.05 (1.17)	2.96, 3.14	33.5
Basic sports medicine	644	3.03 (1.28)	2.93, 3.13	34.6
Ophthalmological conditions	644	2.97 (1.12)	2.88, 3.05	29.2
Genitourinary diseases	644	2.68 (1.12)	2.59, 2.77	19.6
**(c**)				
**Domain/Item**	***N***	**Mean (SD)**	**95% CI**	**% ≥ 4 ^a^**
**Shared Dental Items (both dental roles)**				
Stomatological emergencies	38	3.61 (1.13)	3.23, 3.98	52.6
Major emergency first aid	38	3.53 (1.11)	3.16, 3.89	52.6
Management of clinical workflows in the dental office	38	3.37 (1.02)	3.03, 3.71	39.5
**Health Education (both dental roles)**				
Prophylaxis of dental caries and dento-maxillary anomalies	38	3.79 (1.02)	3.45, 4.12	60.5
Dietary hygiene	38	3.50 (1.08)	3.14, 3.86	50
Healthy lifestyle	38	3.45 (1.03)	3.11, 3.79	39.5
Oral hygiene	38	3.39 (1.08)	3.04, 3.75	39.5
**Dental Physicians**				
Detection of pre-cancerous lesions	27	3.30 (1.23)	2.81, 3.78	51.9
Therapy for harmful oral habits	27	3.22 (0.93)	2.85, 3.59	25.9
Treatment of oro-dental lesions	27	3.15 (1.13)	2.70, 3.60	33.3
Minor surgical interventions	27	2.96 (1.22)	2.48, 3.45	33.3
Oro-dental treatment planning	27	2.89 (1.15)	2.43, 3.35	25.9
Detection of oro-dental conditions	27	2.85 (1.06)	2.43, 3.27	22.2
Orthodontic needs assessment and referral	27	2.85 (1.20)	2.38, 3.33	22.2
Dento-maxillary examination	27	2.74 (1.13)	2.29, 3.19	25.9
**Dental Nurses ^c^**				
Dental prophylaxis procedures	11	3.36 (1.21)	2.55, 4.17	45.5
Assisting in odontological procedures	11	3.09 (1.22)	2.27, 3.91	36.4
Assisting in surgical procedures	11	2.73 (1.35)	1.82, 3.63	27.3

**Notes.** Likert scale: 1 = Very Low Need to 5 = Very High Need. Items grouped by domain; within domain, ranked by mean (descending). Domains ordered by mean of the domain block. ^a^ Percentage of respondents rating the item 4 (High) or 5 (Very High). ᵇ Four Public Health items were administered to all professional roles. ^†^ Worded as active surveillance of children with chronic oro-dental conditions in the dental questionnaire blocks. (b) *n* = 644 reflects respondents identifying as a generalist school physician or generalist school nurse; these items were administered in the generalist block of the questionnaire. (c) Subeligibility blocks reflect the survey skip logic. The *N*, Mean (SD), 95% CI and % ≥ 4 columns pool the two dental roles for the blocks administered to both; role-specific means and item-level *n* are shown in Figure 2. Subgroups are ordered by subgroup *n* (largest first); within each block, items are ranked by mean (descending). ^c^ Estimates from the Dental Nurse subgroup (*n* = 11) are based on very small subgroup sizes and have wide uncertainty intervals. Rows in bold are domain headings within each panel.

### 3.4. Domain-Level Scores, Internal Consistency, and Dimensionality

Internal consistency of the multi-item scales administered to all or generalist respondents was high to excellent (Table 4). Cronbach’s α ranged from 0.872 (Public Health common items, 4 items, *n* = 683) to 0.962 (Ethics, 2 items, *n* = 683). McDonald’s ω, computable for scales with three or more items, was concordant with α throughout, ranging from 0.877 to 0.951. For the dental subdomains, reliability was acceptable for the Dental Physicians subscale (α = 0.888, 8 items, *n* = 27) and the shared Clinical—Dental items (α = 0.831, 2 items, *n* = 38), but lower and less precisely estimated for the Dental Nurses subscale (α = 0.685, 3 items, *n* = 11), with a 95% bootstrap CI extending across zero. The role-specific health-education scales were internally consistent in both groups (generalist items: α = 0.898, 4 items, *n* = 644; dental items: α = 0.928, 4 items, *n* = 38). The 17 items administered to all professional roles were factorable (Kaiser–Meyer–Olkin = 0.917; Bartlett’s χ^2^(136) = 10,622.6, *p* < 0.001). Horn’s parallel analysis supported two factors, a broad general training-need factor and a distinct digital-skills factor (inter-factor r = 0.52). The five-factor solution corresponding to the a priori domains, an exploratory and theoretically imposed solution, showed simple structure, with every item loading most strongly on its intended domain and 76.2% of the variance explained (extraction sums of squared loadings); the full solutions are reported in Supplementary Material S2, and neither solution constitutes evidence of construct validity.

### 3.5. Patterns of Training Need by Professional Role

Item-level mean ratings across all 59 training-need items, by professional role, are visualised in Figure 1 and Figure 2. Three complementary analyses tested for cross-role and cross-stratum differences in domain-level scores: Kruskal–Wallis tests across the four professional roles for each domain; Mann–Whitney U tests of domain scores by sex, urban or rural domicile, and prior training participation; and a multivariable linear regression of the overall mean training-need score on profession, sex, age category, years of experience, domicile, and prior training (Supplementary Material S2).

Across the five domains administered to all four professional roles, statistically significant cross-role differences were detected for digital skills (H(3) = 15.35, *p* = 0.002, ε^2^ = 0.023), ethics (H(3) = 9.82, *p* = 0.020, ε^2^ = 0.014), and social and child rights (H(3) = 8.72, *p* = 0.033, ε^2^ = 0.013). No significant cross-role differences were detected for communication (H(3) = 1.12, *p* = 0.773) or the public health common items (H(3) = 1.98, *p* = 0.576). Among the three domains administered only to generalist physicians and nurses, the clinical (H(1) = 12.23, *p* < 0.001, ε^2^ = 0.019) and health education (H(1) = 12.00, *p* < 0.001, ε^2^ = 0.019) domains differed between professions, while the public health (general-HCP) domain did not (H(1) = 0.30, *p* = 0.586). The underlying item-level pattern is visible in Figure 1 and Figure 2: generalist physicians reported higher mean ratings than generalist nurses on most clinical, public health, and digital skills items (Figure 1), while dental personnel showed mixed estimates with wider uncertainty given the small subgroup sizes (Figure 2; item-level *n* = 11 to 27 by dental role). Descriptively, the largest role divergence within the common block concerned sterilisation and disinfection of instruments and equipment, rated lowest among the common items by generalist physicians and nurses (means 2.91 and 2.83) but 3.71 by dental physicians (their second-highest common item) and 3.73 by dental nurses (their highest); this item-level contrast was not formally tested and is exploratory given the small dental subgroups. All significant effect sizes were small (ε^2^ ≤ 0.023).

No domain showed significant differences in training-need scores by sex, urban or rural domicile, or prior training participation (all *p* > 0.10; rank-biserial |r| ≤ 0.203 across the 24 Mann–Whitney U contrasts). Full output is reported in Supplementary Table S2.1. A multivariable linear regression of the overall mean training-need score on profession, sex, age category, years of experience, domicile, and prior training (*n* = 680; three respondents lacked a codable years-of-experience value) identified profession as the only covariate with a statistically significant contrast: generalist physicians scored 0.25 points higher on the 5-point scale than generalist nurses (B = 0.25, 95% CI 0.08 to 0.43, *p* = 0.005). The dental-physician contrast was not statistically significant and is in any case exploratory given the small subgroup (B = 0.17, 95% CI −0.18 to 0.52, *p* = 0.349; *n* = 28). No statistically significant associations were detected for sex, age category, years of experience, domicile, or prior training. The overall regression model was not statistically significant (adjusted R^2^ = 0.005, F(8, 671) = 1.44, *p* = 0.177). Full output is reported in Supplementary Table S2.2. A sensitivity analysis excluding the dental subgroup, in which all reliability and item-level analyses were re-run on the *n* = 644 generalist sample, yielded effectively unchanged reliability coefficients (Δα ≤ 0.005) and item-level means (Δmean ≤ 0.05 across the 17 items administered to all professional roles). Full comparisons are reported in Supplementary Table S2.3.

### 3.6. Additional Training Topics Suggested

Of the 683 respondents in the analytic sample, 195 (28.6%) provided a valid free-text suggestion to the open-ended question on additional training topics. The five most frequently mentioned theme groups, accounting for 118 of the 195 responses (60.5%), were specific clinical conditions and procedures (*n* = 27; 13.8%); legislation, rights, and professional roles and responsibilities (*n* = 25; 12.8%); child and adolescent psychology and mental health (*n* = 24; 12.3%); emergencies, first aid, and acute care (*n* = 23; 11.8%); and dietary investigation, menus, nutrition, and dietetics (*n* = 19; 9.7%). Communication in specific professional contexts accounted for a further 13 responses (6.7%), collaboration with parents, colleagues, institutions, and other specialities for 12 (6.2%), and addictions, drug use, and risk behaviours for 11 (5.6%).

Smaller but recurrent themes included digital skills and information technology (*n* = 8; 4.1%); Health education, including sexual education (*n* = 7; 3.6%); and children with special educational needs (*n* = 3; 1.5%). The remaining 23 responses (11.8%) covered other specific topics: dental-specific clinical suggestions (*n* = 7), workforce stress management and burnout prevention (*n* = 3), complementary and natural therapies (*n* = 2), adult-trainer certification (*n* = 2), the systemic role of epidemiological triage (*n* = 2), and single mentions of practical skills, conference preparation, school medical office management, pupil time management, cardiovascular health programmes, English-language training, and international exchange in school medicine.

## 4. Discussion

This study presents a national-scale needs assessment of the Romanian school medicine workforce, the largest conducted to date, characterising both current engagement in continuing education and self-reported training priorities across seven thematic domains. The picture is of a motivated workforce (99.0% interested in further training) with clearly articulated self-reported needs and a strong preference for blended, applied delivery. In the multivariable regression, the only covariate showing a statistically significant contrast was profession, with generalist physicians scoring higher than generalist nurses on the overall mean training-need score; no statistically significant associations were detected for sex, age category, years of experience, domicile, or prior training. The explanatory power of the model was negligible (adjusted R^2^ = 0.005; overall *p* = 0.177), so this null result does not establish demographic homogeneity and cannot rule out meaningful subgroup differences, particularly among the small dental subgroups. The similarity of the descriptive priority rankings across strata is nonetheless compatible with a common national CPD core, proposed here as a policy option, with role-specific differentiation for the domains in which significant professional-role differences were observed.

Our findings sit squarely within the WHO global framework for school health services, which positions the school workforce as central to delivering an integrated menu of 87 promotion, prevention, care, and treatment interventions [1,2,4]. In the WHO global SHS Survey, experts identified the highest-priority interventions for routine SHS provision as those relating to health promotion, health education, immunisation, screening, and general care [5]. Our results add a workforce-side perspective to this benchmark: the domains in which international evidence supports intervention are precisely those in which Romanian school medical personnel report feeling underprepared. The MOCHA mapping documented analogous heterogeneity in SHS workforce training, with specific school-medicine training not required for entry in 20 of 30 EU and EEA countries [13]. Heterogeneous school-health workforce resourcing, screening practices that outpace their evidence base, and underresourced psychosocial prevention and mental health [13,14,17,70] apply directly to the Romanian context and motivate the modular, evidence-aligned design pursued by the MED4SCHOOL programme.

### 4.1. Principal Findings

The highest mean training needs were reported for “Emotional and school-adjustment disorders” (3.72; 59% high or very high need) and “Major emergency first aid” (3.70; 58%), with concurrent historical participation already high in emergency management (70.6%) and child-specific pathology (68.5%). The preference for a blended delivery format (50.2%) and for applied learning methods (practical simulations, 63.4%, clinical-case analysis, 44.9%) provides a clear preference profile for the design of capacity-building interventions. This profile is consistent with evidence that blended learning is at least as effective as, and, for knowledge outcomes, often superior to, single-modality formats and that clinicians value hybrid delivery [71,72,73]. Modules addressing major emergencies or the management of emotional disorders would benefit from interactive, scenario-based approaches; simulation-based training can improve clinical skill acquisition and retention, and the limited evidence on skill decay after simulation training argues for periodic refresher practice [74,75,76]. Internal-consistency coefficients (α 0.87–0.96; McDonald’s ω 0.88–0.95) indicate that the instrument was internally consistent in this sample; internal consistency is not evidence of validity, and the five-factor structure was imposed on theoretical grounds while parallel analysis supported two factors, so independent psychometric validation remains to be undertaken.

### 4.2. Mental-Health Competencies

The top-rated training need, “Emotional and school-adjustment disorders” (59.3% high or very high), is consistent with international evidence on the school nursing role in mental health. School nurses report that approximately one-third of their time and student visits relate to mental-health concerns, yet they describe inadequate training to address them [77,78], and evidence syntheses indicate that mental-health training for school nurses improves knowledge, confidence, empowerment, and perceived preparedness to address student needs [78,79]. Evidence on brief, modular delivery is directly relevant to a programme operating under time and cost constraints. A brief interactive web-based programme (“At-Risk”) for primary-school staff improved staff self-efficacy and in-school support for identified pupils [80]; a structured in-service training and implementation-support programme (MH-TIPS) is designed to build assessment, therapeutic-interaction, and referral skills among school nurses [81,82]; and a two-hour CBT-based toolbox has proved acceptable for managing common problems such as sleep and anxiety [83]. In a feasibility pilot, staff who completed MHFA training scored better than untrained colleagues on knowledge, attitudes, confidence and application of helping steps [84], and a school-nurse-delivered brief intervention was associated with reduced student anxiety in a pilot randomised trial [85].

Beyond population-level mental-health promotion, our free-text suggestions highlighted a related cluster of training needs around special-needs and neurodevelopmental conditions (autism spectrum disorder, attention-deficit/hyperactivity disorder, and sensory-processing disorders). Qualitative and practice-oriented school-nursing literature identifies central roles in supporting students with neurodevelopmental disorders, implying corresponding training needs in person-centred, neurodevelopment-informed assessment and counselling [86,87,88]. Given the documented heterogeneity in delivery models across European schools [13,17], brief structured evidence-based modules, adapted to the Romanian scope of practice and embedded in the SDCYC-delivered CPD pathway, are the most defensible entry point for investment in this domain. Romania-specific intervention studies in school mental health remain scarce, so the international evidence must be applied with attention to differences in language, scope of practice, and resources. Several features of the Romanian school setting plausibly amplify this need: school medical staff are often the only health professionals on site, school-psychology coverage is uneven, and the post-pandemic rise in child and adolescent mental-health problems increases the volume and complexity of presentations reaching the school medical office.

### 4.3. Nutrition, Healthy Lifestyle, and Broader Health-Education Content

Nutrition-related items ranked high across both quantitative means (Principles of healthy nutrition: mean 3.62; Healthy nutrition education, generalist personnel: mean 3.58) and open-ended suggestions (the Dietary investigation, menus, nutrition, and dietetics theme accounted for 9.7% of free-text responses, fifth overall). These priorities have direct operational significance in Romania, where school medical staff hold statutory responsibilities for menu assessment and food safety in collectivities and where national surveillance continues to document a high prevalence of childhood overweight. The umbrella review by O’Brien and colleagues indicates that school-based nutrition interventions can positively shift some dietary outcomes, notably fruit and vegetable intake [89], with environment-focused and multicomponent approaches appearing most effective [90], and subsequent reviews indicate that reductions in overweight and obesity risk are modest and most likely when education is combined with physical-activity promotion, parental involvement, and school food-environment change [91,92].

### 4.4. Communication and Family-Centred Care

Three of the highest-rated items concerned communication, with parents, with children by age group, and with authorities, each rated as high or very high need by at least 50% of respondents. Evaluations of nurse-communication training show improvements in communication skills, including empathic responding to parents in emotionally difficult encounters [93,94], and a recent systematic review of communication-skills training for paediatric healthcare professionals caring for children with life-limiting conditions reports behavioural improvements in clinician practice, although effects on child outcomes remain uncertain and trials of brief formats are ongoing [95,96]. Triadic communication (clinician, parent, child) is underrepresented in Romanian curricula. In one observational study of 28 paediatric consultations, children contributed 13% of the interaction, and interruptions, technical language and provider–parent coalitions were identified as barriers to their participation [97], and meta-synthesis evidence on paediatric nurses’ communication with children shows the need for competencies in balancing dyadic and triadic communication [98]. Family-centred-care training modules would address this gap [99]; a scoping review maps a wide array of such programmes, chiefly targeting communication and relational competencies through simulation and role-play, although most have been evaluated only at the level of learner knowledge and confidence [99]. Communication items were rated above the scale midpoint in all four professional roles (Figure 1; means 3.27 to 3.79), which points to a shared, cross-role training priority; the absence of a significant cross-role difference in the communication domain (Kruskal–Wallis *p* = 0.773) is consistent with this pattern but does not by itself establish equivalence between roles. In Romania, school medical staff operate at the interface of schools, families, primary care, and county public health directorates without a formalised communication framework, which makes communication and interinstitutional collaboration core operational competencies. The related theme of interinstitutional collaboration emerged independently in the free-text responses (6.2% of valid responses), indicating that the workforce views communication and collaboration as inseparable.

### 4.5. Barriers, the Predominance of Free-of-Charge Participation, and Digital Skills

Time and schedule constraints (39.8%), cost (37.5%), and the limited local availability of training events (36.6%) were the most-cited obstacles, a ranking that the examples given in the question may have shaped (Section 4.8). The McNemar analysis of historical course attendance provides an important quantitative anchor for the cost finding: for every one of the eight course topics examined, the proportion of respondents who attended free-of-charge sessions was significantly higher than the proportion who paid out of pocket (all *p* < 0.001 after Bonferroni correction), with the asymmetry largest for emergencies and first aid and smallest for digitalisation and innovation (Section 3.2). Together, the cost barrier and the McNemar pattern indicate that historical CPD participation has depended on subsidised or institutionally funded provision rather than on individual outlay; this supports minimising financial barriers in programme design, although a causal effect of free-of-charge delivery on participation cannot be established from these data, and the observed asymmetry may partly reflect the relative supply and accessibility of free versus paid provision, which were not measured. Comparatively low lifetime participation in digitalisation and innovation courses (15.8%) contrasts with moderate-to-high reported need for specific digital competencies, including “Electronic patient records” (mean 3.29) and “Use of generative artificial intelligence assistants” (mean 3.22). Digital-skills needs differed across professional roles (Kruskal–Wallis *p* = 0.002); descriptively, generalist physicians reported higher needs than generalist nurses (median 3.60 vs. 3.00). This pattern is consistent with international consensus that primary-care professionals, including those working with children and adolescents, require integrated digital-health competencies spanning technical, data-security and ethical, and communication dimensions [54,55,100]; the Digital Health Competencies in Medical Education framework provides a competency map adaptable to school medicine [55]. High reported need, low historical participation, and persistent cost and time barriers point to digital skills as an underserved domain addressable through web-based or blended modules.

### 4.6. Relevance, Contextualisation, and the Romanian Policy and Structural Environment

The 12.6% of respondents citing relevance or adaptation of existing courses as a barrier point to the need to tailor training content to Romanian school-medicine practice. The open-ended suggestions, which clustered around legislative clarifications, practical nutrition protocols, interinstitutional collaboration strategies, and operational tools, align with the MED4SCHOOL project’s objective of developing context-specific tools and personnel training. They also reflect a regulatory framework in continuing evolution, redefined in 2023 and renewed again in January 2026 [38,39,40,41,42]. That legislation, rights, and professional roles were the second most frequent free-text theme (12.8% of valid responses) signals that role clarity is itself a perceived training need and argues for coupling CPD with operational tools that anchor the legislative changes in daily practice. Cross-European comparative studies of Health-Promoting Schools (HPSs) implementation show that countries with national-level dissemination and multiple HPS core components report higher implementation coverage and quality [101].

Romania’s broader health-workforce context of out-migration, maldistribution, and an ageing professional cohort [49,50,51] frames these findings: the age structure of the school medicine workforce, with 48.0% aged 50 years or older, mirrors this pattern and makes sustained investment in CPD a retention and quality-improvement lever. Better access to supported CPD and clear development opportunities is consistently associated with higher job satisfaction, better well-being, and lower intention to leave [102,103,104,105]. Conversely, insufficient support for professional development is a notable source of dissatisfaction and stress, and burnout among European hospital clinicians has been documented at around one quarter in cross-national surveys, with organisational-level interventions, particularly changes to working time and task organisation, showing the strongest evidence of benefit [106,107,108]. Although direct burnout-prevalence data for the Romanian school-medicine workforce are not yet available, the free-text responses to our survey surfaced a small but consistent strand on workforce wellbeing, stress, and burnout prevention (1.5% of valid responses) alongside the perceived undervaluation of the school medicine cadre in the wider health system, suggesting that workforce-condition and recognition policies belong in any sustainable capacity-building programme.

### 4.7. Strengths

Key strengths include national geographic coverage (all eight development regions, and 36 of the 41 counties plus Bucharest, reached through the SDCYC communication network), the large overall sample size (*N* = 683 consenting respondents) relative to the estimated size of the Romanian school-medicine workforce, and a role-specific questionnaire structure in which the 59 training-need items were organised into role-defined blocks, with all item-level estimates reported within professional role. Internal consistency was high across the multi-item domains (α 0.87–0.96), and a sensitivity analysis excluding the dental subgroup (|Δα| ≤ 0.005; |Δmean| ≤ 0.05 across the 17 items administered to all professional roles) left the main reliability and item-level findings unchanged. The needs assessment was conducted ahead of, and is now informing, the design of the MED4SCHOOL national capacity-building programme.

### 4.8. Limitations

Several limitations should be considered when interpreting these findings. First, participants were recruited through SDCYC communication channels (the association website, journal, and congress mailing list), which reach, but are not limited to, the association’s membership; because the number of eligible professionals reached cannot be determined, no response rate could be calculated, and professionals reached through these channels may differ systematically from those not reached in motivation, training history, and self-perceived needs (selection bias); the sample therefore reflects the national-scale reach of these channels, not a representative national sample. Second, the sample was overwhelmingly female (97.4%), limiting any analysis by sex but consistent with the documented sex composition of the Romanian school-medicine workforce. Third, regional representation, although spanning all eight development regions, was concentrated in the North-East, North-West, and Centre regions (combined 67.0%), with underrepresentation of the South-Muntenia and South-West Oltenia regions. Fourth, all measures were self-reported, including need ratings; self-reported need may not align with objectively measured competency gaps. Because all items were collected with a single instrument in a single sitting, common-method variance may inflate internal-consistency and interdomain correlations, and participation histories relied on two-year recall without verification against records. Fifth, the cross-sectional design cannot characterise change over time or evaluate the effect of training interventions. Sixth, although the questionnaire was developed for the specific context of Romanian school medicine and shows acceptable internal consistency, it has not been independently psychometrically validated. Seventh, the dental subsample (*N* ≤ 39 across subdomains) is too small for confident inference about dental-specific needs; the dental priorities identified in Table 3c (caries and dento-maxillary prophylaxis, stomatological emergencies, major emergency first aid in dental contexts) should be regarded as hypothesis-generating rather than confirmatory. Eighth, because the health-education blocks administered to generalist and dental personnel contained different items, health-education needs could not be compared across all four professional roles and are reported separately by group. Ninth, the item- and domain-level contrasts were numerous and are reported without formal correction for multiple comparisons, and interaction effects were not formally tested; the cross-role and subgroup comparisons are therefore hypothesis-generating and should be interpreted accordingly. Tenth, the open-text barriers question listed four examples (high costs, lack of time, topics not suited to needs, lack of locally organised events), which may have prompted respondents towards the categories reported most frequently. Finally, although both free-text items were independently dual-coded in full with intercoder reliability reported (Section 2.7), thematic frequencies remain dependent on the categorisation scheme adopted and should be interpreted as descriptive.

### 4.9. Implications for MED4SCHOOL and for Policy

The MED4SCHOOL national capacity-building programme, funded under the European Social Fund Plus (ESF+) component of Health Programme 2021–2027 [109], was designed as a structured response to the training needs reported by the workforce itself; the present study reports the antecedent needs assessment on which that design drew, not the programme itself.

As exploratory, self-reported evidence, the findings carry the following implications for the MED4SCHOOL national training programme: a modular, blended-delivery curriculum, prioritising emotional and adjustment disorders, emergency management, nutrition and health education, communication competencies, and applied digital skills; delivered free of charge at the point of access to remove the cost barrier; complemented by standardised operational tools (clinical protocols, screening forms, communication templates) that respond to the relevance gap reported by the workforce; and embedded in an evaluation framework that goes beyond outcome measurement to examine how and why change occurs [110,111]. Because the descriptive priorities were broadly shared across demographic strata, a common national core curriculum is a defensible policy option; the regression analysis itself, with negligible explanatory power, neither demonstrates nor excludes subgroup differences and is not the basis for this proposal. The free-of-charge pattern (Section 4.5) adds a complementary argument: removing financial barriers is likely to be a precondition of broad workforce reach, although a causal effect of free-of-charge delivery cannot be established from these data. More broadly, the results argue for sustained national investment in the school-medicine workforce as a component of preventive primary care for children and adolescents, aligned with the WHO global SHS framework [1,5] and integrated with workforce retention, equitable geographic distribution, and the Health-Promoting Schools agenda of the WHO European Region and its implementation-support structures [61,112]. CPD coverage should reach the rural and special-needs catchments where unmet preventive-care needs are largest, in line with the equity priorities of the Romanian school-health and primary-care literature [44,45]. These implications are framed by the Romanian regulatory and financing context; their transferability to other school health systems depends on local scope of practice and funding arrangements.

## 5. Conclusions

This national-scale needs assessment of the Romanian school medicine workforce, the largest conducted to date, provides a quantitative evidence base for the self-reported training needs, delivery preferences, and structural barriers of this workforce. Three findings carry direct implications for capacity-building policy. First, 99.0% of respondents expressed interest in further training, with the highest mean training needs concentrated, among generalist physicians and nurses, in Emotional and school-adjustment disorders (mean 3.72), Major emergency first aid (3.70), and Principles of healthy nutrition (3.62), and, across all professional roles, in Communicating with parents (3.58) and Communicating with authorities (3.57); these priorities are consistent with the intervention domains recommended for routine school health services in the WHO global framework and add a workforce-side perspective, namely that domains prioritised in international guidance are also those in which Romanian school medical personnel report feeling underprepared. Second, internal-consistency coefficients across the multi-item training-need domains were high in the main administration blocks (Cronbach’s α 0.87 to 0.96; McDonald’s ω 0.88 to 0.95), and a multivariable regression detected no statistically significant demographic or experiential associations, although its negligible explanatory power (adjusted R^2^ = 0.005) limits its capacity to exclude subgroup differences; the broadly shared descriptive priorities support a common national curriculum as a policy option, with role-specific differentiation for the domains in which significant professional-role differences were observed (digital skills, ethics, and social and child rights across all roles; clinical and health education between the generalist professions); all significant effect sizes were small (ε^2^ ≤ 0.023). Third, for every one of eight historically attended course topics, free-of-charge participation significantly exceeded paid-attendance participation (McNemar’s test, all *p* < 0.001 after Bonferroni correction for eight contrasts), indicating that historical participation has been concentrated in subsidised provision and supporting delivery at no cost at the point of access, although a causal effect of free-of-charge delivery on workforce reach cannot be established from these cross-sectional data.

Taken together, and read as exploratory, self-reported evidence, these findings point to the following design implications for the MED4SCHOOL national capacity-building programme: a modular, blended-delivery curriculum aligned with the WHO Global School Health Services framework; prioritising mental-health and adjustment competencies, emergency response, applied nutrition and health education, family-centred communication, and digital and AI literacy; delivered free of charge at the point of access; complemented by standardised operational tools (clinical protocols, screening forms, communication templates, age-appropriate menu templates) that respond to the relevance gap reported by the workforce; and embedded in a multilevel evaluation framework that captures change at individual, organisational, and system levels. Particular attention should be given to ensuring CPD coverage reaches the rural and special-needs catchments where unmet preventive-care need is greatest. Elements of this needs-assessment approach may be informative for other Central and Eastern European school health systems facing comparable workforce constraints, although transferability will depend on local scope-of-practice and financing arrangements. More broadly, the study positions sustained investment in school-medicine continuing-professional development as a strategic component of preventive primary care for children and adolescents in Romania, integral to the broader national health-workforce attraction-and-retention agenda and to the European Region’s post-pandemic child-health roadmap.

## Figures and Tables

**Table 1:** Sociodemographic and professional characteristics of respondents, overall and by professional role (*N* = 683).

Characteristic	Overall (*N* = 683)	General Physician (*N* = 150)	General Nurse (*N* = 494)	Dental Physician (*N* = 28)	Dental Nurse (*N* = 11)
**Sex**					
Female	665 (97.4%)	138 (92.0%)	491 (99.4%)	25 (89.3%)	11 (100.0%)
Male	18 (2.6%)	12 (8.0%)	3 (0.6%)	3 (10.7%)	0 (0.0%)
**Age (years)**					
25–29	7 (1.0%)	1 (0.7%)	6 (1.2%)	0 (0.0%)	0 (0.0%)
30–34	30 (4.4%)	4 (2.7%)	25 (5.1%)	1 (3.6%)	0 (0.0%)
35–39	53 (7.8%)	4 (2.7%)	45 (9.1%)	3 (10.7%)	1 (9.1%)
40–44	84 (12.3%)	15 (10.0%)	64 (13.0%)	4 (14.3%)	1 (9.1%)
45–49	181 (26.5%)	31 (20.7%)	138 (27.9%)	8 (28.6%)	4 (36.4%)
50–54	181 (26.5%)	38 (25.3%)	134 (27.1%)	5 (17.9%)	4 (36.4%)
55–59	119 (17.4%)	38 (25.3%)	74 (15.0%)	7 (25.0%)	0 (0.0%)
60–65	23 (3.4%)	16 (10.7%)	6 (1.2%)	0 (0.0%)	1 (9.1%)
Over 65	5 (0.7%)	3 (2.0%)	2 (0.4%)	0 (0.0%)	0 (0.0%)
**Residence type**					
Urban	584 (85.5%)	140 (93.3%)	406 (82.2%)	27 (96.4%)	11 (100.0%)
Rural	99 (14.5%)	10 (6.7%)	88 (17.8%)	1 (3.6%)	0 (0.0%)
**Work environment**					
Urban (county seat)	580 (84.9%)	129 (86.0%)	417 (84.4%)	23 (82.1%)	11 (100.0%)
Urban (non-county seat)	86 (12.6%)	18 (12.0%)	63 (12.8%)	5 (17.9%)	0 (0.0%)
Rural	17 (2.5%)	3 (2.0%)	14 (2.8%)	0 (0.0%)	0 (0.0%)
**Development region**					
North-East	169 (24.7%)	23 (15.3%)	134 (27.1%)	10 (35.7%)	2 (18.2%)
South-East	71 (10.4%)	7 (4.7%)	56 (11.3%)	2 (7.1%)	6 (54.5%)
South-Muntenia	28 (4.1%)	7 (4.7%)	20 (4.0%)	1 (3.6%)	0 (0.0%)
South-West Oltenia	55 (8.1%)	22 (14.7%)	30 (6.1%)	3 (10.7%)	0 (0.0%)
West	50 (7.3%)	12 (8.0%)	37 (7.5%)	1 (3.6%)	0 (0.0%)
North-West	155 (22.7%)	46 (30.7%)	105 (21.3%)	2 (7.1%)	2 (18.2%)
Centre	134 (19.6%)	24 (16.0%)	106 (21.5%)	4 (14.3%)	0 (0.0%)
Bucharest-Ilfov	21 (3.1%)	9 (6.0%)	6 (1.2%)	5 (17.9%)	1 (9.1%)
**Years of experience**					
0–4 years	169 (24.9%)	37 (24.8%)	126 (25.6%)	5 (17.9%)	1 (9.1%)
5–9 years	145 (21.3%)	21 (14.1%)	116 (23.6%)	5 (17.9%)	3 (27.3%)
10–14 years	90 (13.2%)	16 (10.7%)	69 (14.0%)	2 (7.1%)	3 (27.3%)
15–19 years	95 (14.0%)	11 (7.4%)	76 (15.4%)	8 (28.6%)	0 (0.0%)
20–24 years	87 (12.8%)	28 (18.8%)	52 (10.6%)	4 (14.3%)	3 (27.3%)
25–29 years	69 (10.1%)	28 (18.8%)	37 (7.5%)	3 (10.7%)	1 (9.1%)
30–34 years	21 (3.1%)	5 (3.4%)	15 (3.0%)	1 (3.6%)	0 (0.0%)
35+ years	4 (0.6%)	3 (2.0%)	1 (0.2%)	0 (0.0%)	0 (0.0%)
Missing	3 (0.4%)	1 (0.7%)	2 (0.4%)	0 (0.0%)	0 (0.0%)
**School unit type served ^a^**					
Nursery	118 (17.3%)	35 (23.3%)	83 (16.8%)	0 (0.0%)	0 (0.0%)
Kindergarten	358 (52.4%)	113 (75.3%)	230 (46.6%)	11 (39.3%)	4 (36.4%)
Primary school	222 (32.5%)	92 (61.3%)	114 (23.1%)	12 (42.9%)	4 (36.4%)
Lower secondary school	322 (47.1%)	114 (76.0%)	186 (37.7%)	16 (57.1%)	6 (54.5%)
Vocational school	80 (11.7%)	37 (24.7%)	41 (8.3%)	1 (3.6%)	1 (9.1%)
Special-needs school	34 (5.0%)	12 (8.0%)	22 (4.5%)	0 (0.0%)	0 (0.0%)
High school	287 (42.0%)	107 (71.3%)	151 (30.6%)	20 (71.4%)	9 (81.8%)
University	17 (2.5%)	9 (6.0%)	4 (0.8%)	4 (14.3%)	0 (0.0%)

**Notes.** Values are *n* (%) of column total unless otherwise stated. Column percentages may not sum to 100 because of rounding. Percentages for years of experience are valid percentages, computed on respondents with non-missing data (*n* = 680 overall; 149 general physicians; 492 general nurses). ^a^ Multiselect question: respondents could report working in more than one type of school unit, so column percentages sum to more than 100%. Rows in bold are group headings.

**Table 2:** Self-reported participation in continuing-education activities, by event type and course topic (*N* = 683).

Category	*n*	%
**Event types attended (any prior participation) ^a^**		
Courses	604	88.4
Physical congresses	330	48.3
Online congresses	306	44.8
Workshops	237	34.7
Webinars	237	34.7
Other events	224	32.8
Symposia	220	32.2
**Course topics attended (any prior participation) ^a^**		
Emergencies and first aid	482	70.6
Child pathology	468	68.5
Health education	352	51.5
Hygiene and disinfection	275	40.3
Communication with patients	261	38.2
Patient rights	211	30.9
Ethics	159	23.3
Digitalisation and innovation	108	15.8
**Barriers to participation in continuing-education activities ^a^**		
Time/schedule constraints	272	39.8
Cost	256	37.5
Limited local availability of training events	250	36.6
Relevance or adaptation of available content	86	12.6
Organisational or employer issues	21	3.1
Information or communication gaps	7	1.0
Format of training events	7	1.0
Specific personal circumstances	6	0.9
No barriers reported	36	5.3
No substantive answer ^b^	14	2.0

**Notes.** Values are absolute counts (*n*) and column percentages (%) of the consenting analytic sample (*N* = 683). ^a^ Questions allowed for multiple responses: respondents could indicate participation in several event types, multiple course topics, and various barrier categories. Column percentages, therefore, do not sum to 100%. The “No barriers reported” and “No substantive answer” rows are each mutually exclusive with all other rows by definition (respondents in these rows endorsed no barrier category). ^b^ Responses consisting only of a placeholder character (“-” or “/”; *n* = 9), “do not know” (*n* = 2), or an off-topic remark (*n* = 3). Abbreviations: CPD, continuing professional development. Rows in bold are group headings (event types, course topics, barriers).

**Table 4:** Domain-level mean training-need ratings and internal-consistency statistics, by sample-restriction block (5-point Likert scale; 1 = Very Low Need to 5 = Very High Need).

Domain	Items (k)	*n*	Mean (SD)	Cronbach α (95% CI) ^a^	McDonald ω ^b^
**Domains administered to all professional roles (all HCPs)**					
Communication	4	683	3.55 (1.12)	0.951 (0.941–0.959)	0.951
Social and Child Rights	2	683	3.44 (1.11)	0.901 (0.876–0.923)	n/a
Ethics	2	683	3.24 (1.27)	0.962 (0.951–0.972)	n/a
Digital Skills	5	683	3.20 (1.12)	0.911 (0.895–0.924)	0.911
Public Health (common items) ^†^	4	683	3.17 (1.06)	0.872 (0.851–0.891)	0.877
**Domains administered to generalist physicians and nurses only (general HCPs)**					
Health Education	4	644	3.44 (1.15)	0.898 (0.877–0.914)	0.901
Public Health (general-HCP items)	8	644	3.40 (1.06)	0.942 (0.933–0.950)	0.942
Clinical (general HCPs)	12	644	3.25 (0.88)	0.932 (0.921–0.940)	0.933
**Subdomains administered to dental personnel only**					
Clinical—Dental (shared items)	2	38	3.57 (1.03)	0.831 (0.645–0.939)	n/a
Health Education—Dental	4	38	3.53 (0.96)	0.928 (0.864–0.965)	0.930
Public Health—Dental (single item)	1	38	3.37 (1.02)	n/a	n/a
Clinical—Dental Nurses	3	11	3.06 (0.99)	0.685 (−0.504–0.955)	0.759
Clinical—Dental Physicians	8	27	3.00 (0.85)	0.888 (0.724–0.949)	0.888

**Notes.** Within each sample-restriction block, rows are ordered by domain mean (highest to lowest). Sample-restriction block headings (rows in bold) indicate the survey skip-logic group to which the domain was administered. ^a^ Cronbach’s α with 95% bootstrap confidence interval (1000 resamples). ᵇ McDonald’s ω was computed only for scales with three or more items; reported as “n/a” for two-item and single-item scales for which the model is not identified. Mean interitem correlations ranged from 0.417 (Clinical—Dental Nurses) to 0.928 (Ethics). ^†^ The four public-health items administered to all professional roles (Table 3a); one of them is worded as active surveillance of children with chronic oro-dental conditions in the dental questionnaire blocks. Abbreviations: HCP, healthcare professional; SD, standard deviation; CI, confidence interval.

## Data Availability

The data presented in this study are available on request from the corresponding author. Public deposition of the individual-level dataset is not possible because the participants’ informed consent (ethics approval DEP122/26.03.2025, “Iuliu Hațieganu” University of Medicine and Pharmacy Cluj-Napoca) does not extend to public sharing of individual records and because the respondents form a small, well-defined national professional community in which combinations of profession, region, and demographic characteristics carry a realistic risk of deductive re-identification even after de-identification. Requests are honoured subject to the consent terms and Regulation (EU) 2016/679 (GDPR).

## Supplementary Materials

The following supporting information can be downloaded at: https://www.mdpi.com/article/10.3390/healthcare14193245/s1, Supplementary Material S1: Survey Instrument (English Translation); Supplementary Material S2: Auxiliary analyses (Mann–Whitney tests by sex, residence type, and prior training; multivariable regression; sensitivity analyses excluding the dental subgroup; and exploratory factor analysis of the 17 items administered to all professional roles); Supplementary Material S3: Category definitions and example statements for the codebooks used in the content analysis of the two open-ended items.

## Abbreviations

The following abbreviations are used in this manuscript:

α	Cronbach’s alpha (internal-consistency reliability coefficient)
AI	artificial intelligence
CBT	cognitive behavioural therapy
CEE	Central and Eastern European
CI	confidence interval
CME	continuing medical education
COSI	Childhood Obesity Surveillance Initiative (WHO European Region)
CPD	continuing professional development
EEA	European Economic Area
ε^2^	epsilon-squared (effect-size measure for the Kruskal–Wallis test)
ESF+	European Social Fund Plus
EU	European Union
GDPR	General Data Protection Regulation (EU Regulation 2016/679)
HBSC	Health Behaviour in School-aged Children study
HC3	heteroscedasticity-consistent (type 3) robust standard errors
HCP	healthcare professional
HPS	Health-Promoting School
IQR	interquartile range
LLM	large language model
MED4SCHOOL	Continuous Training in School Medicine, the Foundation of a Healthy Generation (Romanian translation: Formarea continuă în medicina școlară, fundația unei generații sănătoase) (Romanian EU-funded national capacity-building project, SMIS code 348094)
MHFA	Mental-health first aid
MOCHA	Models of Child Health Appraised (European consortium)
MoE	Ministry of Education
MoH	Ministry of Health
ω	McDonald’s omega (internal-consistency reliability coefficient)
SD	standard deviation
SHS	school health service
SDCYC	Society of Doctors from Children’s and Youth Collectivities
SMIS	Single Management Information System (Romanian identifier code for EU-funded projects)
SPSS	Statistical Package for the Social Sciences (IBM SPSS Statistics, version 31.0 for macOS)
STI	sexually transmitted infection
STROBE	Strengthening the Reporting of Observational Studies in Epidemiology
VIF	variance inflation factor
WHO	World Health Organization
YRBSS	Youth Risk Behaviour Survey
χ^2^	chi-square (test statistic)
