# Supplementary material for: Self-Reported Training Needs and Continuing-Education Preferences of the School Medicine Workforce in Romania: A National Cross-Sectional Survey
Source: Healthcare (Basel). 2026 Oct 1;14(19):3245. doi: 10.3390/healthcare14193245 (PMC13664956; doi:10.3390/healthcare14193245)
Supplement: Supplementary file 1 [file healthcare-14-03245-s001.zip › 2026.09.24_healthcare-4558549_S1_R3.pdf]

Supplementary Material S1. Survey Instrument (English Translation)

Part I. Demographic information

1. Age (years). *

- ☐ 25–29
- ☐ 30–34
- ☐ 35–39
- ☐ 40–44
- ☐ 45–49
- ☐ 50–54
- ☐ 55–59
- ☐ 60–65
- ☐ Over 65

2. Sex. *

- ☐ Female
- ☐ Male

3. Your place of residence is in a: *

- ☐ Urban area
- ☐ Rural area

4. Your place of residence is in the county of: *

Dropdown listing all 41 Romanian counties and the municipality of Bucharest (Alba, Arad, Argeş, Bacău, Braşov, Brăila, Bihor, Bistriţa, Botoşani, Bucureşti, Buzău, Caraş-Severin, Călăraşi, Cluj, Constanţa, Covasna, Dâmboviţa, Dolj, Galaţi, Giurgiu, Gorj, Harghita, Hunedoara, Ialomiţa, Iaşi, Ilfov, Maramureş, Mehedinţi, Mureş, Neamţ, Olt, Prahova, Satu-Mare, Sălaj, Sibiu, Suceava, Teleorman, Timiş, Tulcea, Vaslui, Vâlcea, Vrancea).

Part II. Professional activity

5. Your workplace is located in a: *

- ☐ Urban area (county-seat city)
- ☐ Urban area (city that is not a county seat)
- ☐ Rural area

6. Locality where you carry out your school-medicine activity. *

Free text.

7. How many years have you been practising in a school medicine office? *

Free text - numeric (years).

8. The types of school / educational units under your responsibility are: *

Tick all that apply.

- ☐ Nursery
- ☐ Kindergarten
- ☐ Primary school
- ☐ Lower secondary school
- ☐ Special needs school
- ☐ Vocational school
- ☐ High school

☐ University

9. Number of pre-schoolers / pupils / students in each educational unit under your responsibility:

Tick multiple options if you serve more than one type of educational unit.

Item	<100	100–300	301–600	601–1,000	1,000–1,500	1,500–2,000	>2,000
Nursery	☐	☐	☐	☐	☐	☐	☐
Kindergarten	☐	☐	☐	☐	☐	☐	☐
Primary school	☐	☐	☐	☐	☐	☐	☐
Lower secondary school	☐	☐	☐	☐	☐	☐	☐
Special needs school	☐	☐	☐	☐	☐	☐	☐
Vocational school	☐	☐	☐	☐	☐	☐	☐
High school	☐	☐	☐	☐	☐	☐	☐
University	☐	☐	☐	☐	☐	☐	☐

10. How many training / continuing-education events have you attended in the last 2 years? *

Tick all that apply.

Item	None	1	2	3	4	5	>5
Courses	☐	☐	☐	☐	☐	☐	☐
Scientific congress / conferences - in-person	☐	☐	☐	☐	☐	☐	☐
Scientific congress / conferences - online	☐	☐	☐	☐	☐	☐	☐
Practical workshops	☐	☐	☐	☐	☐	☐	☐
Webinars	☐	☐	☐	☐	☐	☐	☐
Symposia	☐	☐	☐	☐	☐	☐	☐
Other	☐	☐	☐	☐	☐	☐	☐

11. What were the topics of the courses you attended, and the mode of access?

Tick all that apply.

Item	Free of charge	Fee-based
Emergencies / first aid	☐	☐
Child pathology (respiratory, infectious, digestive, endocrine and metabolic, neurological, orthopaedic conditions)	☐	☐
Hygiene and disinfection rules; medical-waste collection	☐	☐
Health education	☐	☐
Ethics	☐	☐
Patients' / children's rights	☐	☐
Communication with patients	☐	☐
Digitalisation and innovation	☐	☐

Training / training need (introduction to the preference questions below)

12. Are you interested in participating in training in your field of activity? *

- ☐ Yes
- ☐ No

13. What are the main barriers you encounter in participating in professional training / continuing-education events? *

Please describe (e.g., high costs, lack of time, training topics not suited to your needs, lack of opportunities - no events organised in your region, etc.). Free text.

14. How would you prefer these training activities to be delivered? *

- ☐ In-person (on-site)
- ☐ Online (through virtual platforms)
- ☐ Blended (in-person and online)

15. At which university centre would you like to attend training? *

Please indicate a maximum of two options / preferences. Tick all that apply.

- ☐ București
- ☐ Cluj
- ☐ Constanța
- ☐ Craiova
- ☐ Iași
- ☐ Târgu Mureș
- ☐ Timișoara
- ☐ Other (please specify)

16. Which teaching methods would you prefer? *

Tick all that apply.

- ☐ Webinars and interactive online training sessions
- ☐ Clinical case analysis
- ☐ Simulations and practical scenarios
- ☐ Team-based learning
- ☐ Other (please specify)

Part III. Professional category (skip-logic anchor)

17. What is your profession? *

Skip logic: response routes the respondent to the corresponding role-specific block of training-need items below.

- ☐ Physician in a school medical office → Block A (school physician)
- ☐ Nurse in a school medical office → Block B (school nurse)
- ☐ Dental physician in a school dental office → Block C (school dental physician)
- ☐ Nurse in a school dental office → Block D (school dental nurse)
- ☐ Other (please specify)

Part IV - Block A. Training needs of the school physician

For each item below, indicate how strongly you need training for your activity, using the 5-point scale: 1 - Very low; 2 - Low; 3 - Moderate; 4 - High; 5 - Very high.

*A.1. Clinical domain **

Item	1 - Very low	2 - Low	3 - Moderate	4 - High	5 - Very high
First aid in major emergencies	☐	☐	☐	☐	☐
Respiratory pathology; management of seasonal viral infections	☐	☐	☐	☐	☐
Infectious diseases	☐	☐	☐	☐	☐
Genito-urinary diseases	☐	☐	☐	☐	☐
Digestive diseases	☐	☐	☐	☐	☐
Cardiovascular conditions	☐	☐	☐	☐	☐
Endocrinological conditions and diabetes mellitus	☐	☐	☐	☐	☐
Ophthalmological conditions	☐	☐	☐	☐	☐
Neuro-psychiatric conditions	☐	☐	☐	☐	☐
Emotional disorders and school-adjustment difficulties	☐	☐	☐	☐	☐
Postural and vertebral statics disorders	☐	☐	☐	☐	☐
Elementary notions of sports medicine	☐	☐	☐	☐	☐

*A.2. Hygiene and public health **

Item	1 - Very low	2 - Low	3 - Moderate	4 - High	5 - Very high
Legislation governing the functioning of school medical offices	☐	☐	☐	☐	☐
National immunisation programme under special epidemiological conditions	☐	☐	☐	☐	☐
Hygiene of educational establishments and of the teaching-learning process; sanitary conditions, activity-rest regime	☐	☐	☐	☐	☐
Verification of food preparation and serving conditions in children's collectivities; dietary investigation	☐	☐	☐	☐	☐
Principles of healthy nutrition; designing a weekly menu for kindergarten / school	☐	☐	☐	☐	☐
Periodic health examinations of pupils ("bilanț" examinations)	☐	☐	☐	☐	☐
Epidemiological triage in the collectivity	☐	☐	☐	☐	☐
Epidemiological investigation and intervention in an outbreak	☐	☐	☐	☐	☐
Active surveillance of children with chronic conditions	☐	☐	☐	☐	☐
Preparation of current health-information-system reports	☐	☐	☐	☐	☐
Management of medical waste	☐	☐	☐	☐	☐
Sterilisation and disinfection of instruments and equipment	☐	☐	☐	☐	☐

*A.3. Health education and sexual education **

Item	1 - Very low	2 - Low	3 - Moderate	4 - High	5 - Very high
Education for healthy nutrition	☐	☐	☐	☐	☐
Promotion of physical activity	☐	☐	☐	☐	☐
Prevention of health-risk behaviours (tobacco, alcohol, drug use; violent behaviour)	☐	☐	☐	☐	☐
Prevention of adolescent pregnancy, contraception, sexually transmitted infections	☐	☐	☐	☐	☐

*A.4. Social domain / children's rights **

Item	1 - Very low	2 - Low	3 - Moderate	4 - High	5 - Very high
Children's rights	☐	☐	☐	☐	☐
Physically abused, neglected, emotionally abused, and sexually abused child	☐	☐	☐	☐	☐

*A.5. Ethics **

Item	1 - Very low	2 - Low	3 - Moderate	4 - High	5 - Very high
Confidentiality of data	☐	☐	☐	☐	☐
Informed consent (particularities for the minor patient)	☐	☐	☐	☐	☐

*A.6. Digital skills and innovation **

Item	1 - Very low	2 - Low	3 - Moderate	4 - High	5 - Very high
Basic Office skills	☐	☐	☐	☐	☐
Data entry in the national health insurance information system	☐	☐	☐	☐	☐
Patient records in electronic health-information systems	☐	☐	☐	☐	☐
Communication by electronic mail	☐	☐	☐	☐	☐
Use of large language models	☐	☐	☐	☐	☐

*A.7. Communication **

Item	1 - Very low	2 - Low	3 - Moderate	4 - High	5 - Very high
Communication with children (adapted to the particularities of each age group)	☐	☐	☐	☐	☐
Communication with parents	☐	☐	☐	☐	☐
Communication with authorities (school, town hall, Public Health Directorate)	☐	☐	☐	☐	☐
Motivation and persuasion	☐	☐	☐	☐	☐

QA.8. Additional domains - please indicate any other domains or topics you would like to explore further: *

Free text.

Part IV - Block B. Training needs of the school nurse

Identical seven-domain structure to Block A. Items are administered with the same wording and the same 5-point Likert scale (1 - Very low; 5 - Very high), reflecting the shared scope of practice between school physicians and school nurses for these topics.

*B.1. Clinical domain **

Item	1 - Very low	2 - Low	3 - Moderate	4 - High	5 - Very high
First aid in major emergencies	☐	☐	☐	☐	☐
Respiratory pathology; management of seasonal viral infections	☐	☐	☐	☐	☐
Infectious diseases	☐	☐	☐	☐	☐
Genito-urinary diseases	☐	☐	☐	☐	☐
Digestive diseases	☐	☐	☐	☐	☐
Cardiovascular conditions	☐	☐	☐	☐	☐
Endocrinological conditions and diabetes mellitus	☐	☐	☐	☐	☐
Ophthalmological conditions	☐	☐	☐	☐	☐
Neuro-psychiatric conditions	☐	☐	☐	☐	☐
Emotional disorders and school-adjustment difficulties	☐	☐	☐	☐	☐
Postural and vertebral statics disorders	☐	☐	☐	☐	☐
Elementary notions of sports medicine	☐	☐	☐	☐	☐

*B.2. Hygiene and public health **

Item	1 - Very low	2 - Low	3 - Moderate	4 - High	5 - Very high
Legislation governing the functioning of school medical offices	☐	☐	☐	☐	☐
National immunisation programme under special epidemiological conditions	☐	☐	☐	☐	☐
Hygiene of educational establishments and of the teaching-learning process; sanitary conditions, activity-rest regime	☐	☐	☐	☐	☐
Verification of food preparation and serving conditions in children's collectivities; dietary investigation	☐	☐	☐	☐	☐
Principles of healthy nutrition; designing a weekly menu for kindergarten / school	☐	☐	☐	☐	☐
Periodic health examinations of pupils ("bilanț" examinations)	☐	☐	☐	☐	☐
Epidemiological triage in the collectivity	☐	☐	☐	☐	☐
Epidemiological investigation and intervention in an outbreak	☐	☐	☐	☐	☐
Active surveillance of children with chronic conditions	☐	☐	☐	☐	☐
Preparation of current health-information-system reports	☐	☐	☐	☐	☐
Management of medical waste	☐	☐	☐	☐	☐
Sterilisation and disinfection of instruments and equipment	☐	☐	☐	☐	☐

*B.3. Health education and sexual education **

Item	1 - Very low	2 - Low	3 - Moderate	4 - High	5 - Very high
Education for healthy nutrition	☐	☐	☐	☐	☐
Promotion of physical activity	☐	☐	☐	☐	☐
Prevention of health-risk behaviours (tobacco, alcohol, drug use; violent behaviour)	☐	☐	☐	☐	☐
Prevention of adolescent pregnancy, contraception, sexually transmitted infections	☐	☐	☐	☐	☐

*B.4. Social domain / children's rights **

Item	1 - Very low	2 - Low	3 - Moderate	4 - High	5 - Very high
Children's rights	☐	☐	☐	☐	☐
Physically abused, neglected, emotionally abused, and sexually abused child	☐	☐	☐	☐	☐

*B.5. Ethics **

Item	1 - Very low	2 - Low	3 - Moderate	4 - High	5 - Very high
Confidentiality of data	☐	☐	☐	☐	☐
Informed consent (particularities for the minor patient)	☐	☐	☐	☐	☐

*B.6. Digital skills and innovation **

Item	1 - Very low	2 - Low	3 - Moderate	4 - High	5 - Very high
Basic Office skills	☐	☐	☐	☐	☐
Data entry in the national health insurance information system	☐	☐	☐	☐	☐
Patient records in electronic health-information systems	☐	☐	☐	☐	☐
Communication by electronic mail	☐	☐	☐	☐	☐
Use of large language models	☐	☐	☐	☐	☐

*B.7. Communication **

Item	1 - Very low	2 - Low	3 - Moderate	4 - High	5 - Very high
Communication with children (adapted to the particularities of each age group)	☐	☐	☐	☐	☐
Communication with parents	☐	☐	☐	☐	☐
Communication with authorities (school, town hall, Public Health Directorate)	☐	☐	☐	☐	☐
Motivation and persuasion	☐	☐	☐	☐	☐

QB.8. Additional domains - please indicate any other domains or topics you would like to explore further: *

Free text.

Part IV - Block C. Training needs of the school dental physician

Items reflect the dental-specific scope of practice defined by the Methodology approved by Joint Order No. 2.508/4.493/2023 and by Romanian College of Dentists guidance for school dental offices.

*C.1. Clinical domain **

Item	1 - Very low	2 - Low	3 - Moderate	4 - High	5 - Very high
First aid in major emergencies	☐	☐	☐	☐	☐
Dental emergencies	☐	☐	☐	☐	☐
Dento-maxillary examination	☐	☐	☐	☐	☐
Detection of oro-dental conditions	☐	☐	☐	☐	☐
Preparation of treatment plans for oro-dental conditions	☐	☐	☐	☐	☐
Treatment of oro-dental lesions	☐	☐	☐	☐	☐
Minor surgical interventions (extractions, abscess incision)	☐	☐	☐	☐	☐

Item	1 - Very low	2 - Low	3 - Moderate	4 - High	5 - Very high
Assessment of orthodontic needs and referral to specialist dental practice	☐	☐	☐	☐	☐
Implementation of therapy to discontinue oral parafunctional habits	☐	☐	☐	☐	☐
Detection of pre-cancerous lesions of the oral and perioral soft tissues	☐	☐	☐	☐	☐

C.2. Hygiene and public health *

Item	1 - Very low	2 - Low	3 - Moderate	4 - High	5 - Very high
Legislation governing the functioning of school medical offices	☐	☐	☐	☐	☐
Management of functional circuits in the dental office	☐	☐	☐	☐	☐
Active surveillance of children with chronic oro-dental conditions	☐	☐	☐	☐	☐
Management of medical waste	☐	☐	☐	☐	☐
Sterilisation and disinfection of instruments and equipment	☐	☐	☐	☐	☐

C.3. Health education *

Item	1 - Very low	2 - Low	3 - Moderate	4 - High	5 - Very high
Healthy lifestyle	☐	☐	☐	☐	☐
Oral hygiene	☐	☐	☐	☐	☐
Dietary hygiene	☐	☐	☐	☐	☐
Prophylaxis of dental caries and dento-maxillary anomalies	☐	☐	☐	☐	☐

C.4. Social domain / children's rights *

Item	1 - Very low	2 - Low	3 - Moderate	4 - High	5 - Very high
Children's rights	☐	☐	☐	☐	☐
Physically abused, neglected, emotionally abused, and sexually abused child	☐	☐	☐	☐	☐

C.5. Ethics *

Item	1 - Very low	2 - Low	3 - Moderate	4 - High	5 - Very high
Confidentiality of data	☐	☐	☐	☐	☐
Informed consent (particularities for the minor patient)	☐	☐	☐	☐	☐

C.6. Digital skills and innovation *

Item	1 - Very low	2 - Low	3 - Moderate	4 - High	5 - Very high
Basic Office skills	☐	☐	☐	☐	☐

Item	1 - Very low	2 - Low	3 - Moderate	4 - High	5 - Very high
Data entry in the national health insurance information system	☐	☐	☐	☐	☐
Patient records in electronic health-information systems	☐	☐	☐	☐	☐
Communication by electronic mail	☐	☐	☐	☐	☐
Use of large language models	☐	☐	☐	☐	☐

*C.7. Communication **

Item	1 - Very low	2 - Low	3 - Moderate	4 - High	5 - Very high
Communication with children (adapted to the particularities of each age group)	☐	☐	☐	☐	☐
Communication with parents	☐	☐	☐	☐	☐
Communication with authorities (school, town hall, Public Health Directorate)	☐	☐	☐	☐	☐
Motivation and persuasion	☐	☐	☐	☐	☐

QC.8. Additional domains - please indicate any other domains or topics you would like to explore further: *

Free text.

Part IV - Block D. Training needs of the school dental nurse

Items reflect the dental nurse scope of practice (assisting roles, prophylaxis, hygiene, ethics, and communication).

*D.1. Clinical domain **

Item	1 - Very low	2 - Low	3 - Moderate	4 - High	5 - Very high
First aid in major emergencies	☐	☐	☐	☐	☐
Dental emergencies	☐	☐	☐	☐	☐
Assisting the dentist during odontal (restorative) procedures	☐	☐	☐	☐	☐
Assisting the dentist during surgical dental procedures	☐	☐	☐	☐	☐
Performing professional dental-prophylaxis procedures (professional cleaning)	☐	☐	☐	☐	☐

*D.2. Hygiene and public health **

Item	1 - Very low	2 - Low	3 - Moderate	4 - High	5 - Very high
Legislation governing the functioning of school medical offices	☐	☐	☐	☐	☐
Management of functional circuits in the dental office	☐	☐	☐	☐	☐
Active surveillance of children with chronic oro-dental conditions	☐	☐	☐	☐	☐
Management of medical waste	☐	☐	☐	☐	☐

Item	1 - Very low	2 - Low	3 - Moderate	4 - High	5 - Very high
Sterilisation and disinfection of instruments and equipment	☐	☐	☐	☐	☐

*D.3. Health education **

Item	1 - Very low	2 - Low	3 - Moderate	4 - High	5 - Very high
Healthy lifestyle	☐	☐	☐	☐	☐
Oral hygiene	☐	☐	☐	☐	☐
Dietary hygiene	☐	☐	☐	☐	☐
Prophylaxis of dental caries and dento-maxillary anomalies	☐	☐	☐	☐	☐

*D.4. Social domain / children's rights **

Item	1 - Very low	2 - Low	3 - Moderate	4 - High	5 - Very high
Children's rights	☐	☐	☐	☐	☐
Physically abused, neglected, emotionally abused, and sexually abused child	☐	☐	☐	☐	☐

*D.5. Ethics **

Item	1 - Very low	2 - Low	3 - Moderate	4 - High	5 - Very high
Confidentiality of data	☐	☐	☐	☐	☐
Informed consent (particularities for the minor patient)	☐	☐	☐	☐	☐

*D.6. Digital skills and innovation **

Item	1 - Very low	2 - Low	3 - Moderate	4 - High	5 - Very high
Basic Office skills	☐	☐	☐	☐	☐
Data entry in the national health insurance information system	☐	☐	☐	☐	☐
Patient records in electronic health-information systems	☐	☐	☐	☐	☐
Communication by electronic mail	☐	☐	☐	☐	☐
Use of large language models	☐	☐	☐	☐	☐

*D.7. Communication **

Item	1 - Very low	2 - Low	3 - Moderate	4 - High	5 - Very high
Communication with children (adapted to the particularities of each age group)	☐	☐	☐	☐	☐
Communication with parents	☐	☐	☐	☐	☐
Communication with authorities (school, town hall, Public Health Directorate)	☐	☐	☐	☐	☐
Motivation and persuasion	☐	☐	☐	☐	☐

QD.8. Additional domains - please indicate any other domains or topics you would like to explore further: *

Free text.

End of questionnaire

Survey closing footer: "This content is neither created nor endorsed by Google." - default Google Forms footer; not part of the instrument.

Notes on instrument structure as reproduced above:

Items marked with "" were configured as mandatory in the deployed Google Forms instrument.*

The questionnaire was administered in Romanian; the above is an English translation prepared by the bilingual study team for the purpose of journal publication. Item ordering, response scales, and skip-logic match the original.

Skip logic at Q17 routed each respondent to one of four role-specific blocks (A school physician, B school nurse, C school dental physician, D school dental nurse). Within each block, all items were administered to all respondents in that role.
