# Supplementary material for: Self-Reported Training Needs and Continuing-Education Preferences of the School Medicine Workforce in Romania: A National Cross-Sectional Survey
Source: Healthcare (Basel). 2026 Oct 1;14(19):3245. doi: 10.3390/healthcare14193245 (PMC13664956; doi:10.3390/healthcare14193245)
Supplement: Supplementary file 1 [file healthcare-14-03245-s001.zip › 2026.09.24_healthcare-4558549_S2_R3.pdf]

Supplementary Material S2. Auxiliary analyses (Mann–Whitney tests by sex, residence type, and prior training; multivariable regression; sensitivity analyses excluding the dental subgroup; and exploratory factor analysis of the 17 items administered to all professional roles).

Table S2.1. Mann–Whitney U tests of domain-level training-need scores by sex, urban or rural domicile, and prior training participation.

Domain	Comparison	Group 1 (n, Mdn)	Group 2 (n, Mdn)	U	p	|r|
Communication	Female vs. Male	665, 3.50	18, 3.75	5432.0	0.499	0.092
	Urban vs. Rural	584, 3.50	99, 3.75	27116.5	0.319	0.062
	Any prior training vs. None	649, 3.50	34, 4.00	10338.0	0.532	0.063
Social and Child Rights	Female vs. Male	665, 3.50	18, 3.50	5836.5	0.855	0.025
	Urban vs. Rural	584, 3.00	99, 3.50	26151.5	0.121	0.095
	Any prior training vs. None	649, 3.50	34, 3.50	11031.0	0.999	0.000
Ethics	Female vs. Male	665, 3.00	18, 3.00	5309.0	0.403	0.113
	Urban vs. Rural	584, 3.00	99, 3.00	28498.5	0.818	0.014
	Any prior training vs. None	649, 3.00	34, 3.00	10681.5	0.749	0.032
Digital Skills	Female vs. Male	665, 3.00	18, 3.40	5078.0	0.271	0.152
	Urban vs. Rural	584, 3.00	99, 3.20	28008.0	0.619	0.031
	Any prior training vs. None	649, 3.00	34, 3.00	10638.5	0.724	0.036
Public Health (common items)	Female vs. Male	665, 3.00	18, 3.12	5474.5	0.535	0.085
	Urban vs. Rural	584, 3.00	99, 3.25	26917.5	0.271	0.069
	Any prior training vs. None	649, 3.00	34, 3.00	10829.0	0.855	0.018
Health Education	Female vs. Male	629, 3.50	15, 3.75	3988.0	0.303	0.155
	Urban vs. Rural	546, 3.50	98, 3.25	27622.5	0.606	0.032
	Any prior training vs. None	610, 3.50	34, 3.00	10498.0	0.903	0.012
Public Health (General-HCP items)	Female vs. Male	629, 3.38	15, 3.75	4088.0	0.376	0.133
	Urban vs. Rural	546, 3.38	98, 3.62	25048.5	0.314	0.064
	Any prior training vs. None	610, 3.50	34, 3.31	10479.0	0.918	0.011
Clinical (General HCPs)	Female vs. Male	629, 3.17	15, 3.42	3758.0	0.178	0.203
	Urban vs. Rural	546, 3.17	98, 3.33	25278.0	0.384	0.055
	Any prior training vs. None	610, 3.17	34, 3.29	9240.0	0.284	0.109

Notes. Mdn, median domain score on the 5-point Likert scale (1 = Very Low Need to 5 = Very High Need); U, Mann–Whitney U statistic; |r|, absolute rank-biserial correlation as effect size. p-values are two-sided. Prior training denotes attendance of at least one training or continuing-education event in the last 2 years (questionnaire item II.10). Domain scores were computed for respondents answering all items in a domain (complete-case). The Health Education, Public Health (General-HCP items), and Clinical (General HCPs) domains were administered only to generalist school physicians and nurses (n = 644); all other domains were administered to the full consented analytic sample (N = 683). The dental-only sub-domains were not tested

owing to the small dental subgroups (n = 11–39, 92.3% female). No contrast reached statistical significance at $p < 0.05$; all effect sizes were small ($|r| \leq 0.21$).

Table S2.2. Multivariable linear regression of the overall mean training-need score (17 items administered to all professional roles) on demographic and experiential predictors (n = 680).

Predictor	B	SE	95% CI	t	p
(Intercept)	3.517	0.295	2.936, 4.097	11.90	<0.001
General Physician (vs. General Nurse)	0.254	0.090	0.077, 0.431	2.82	0.005
Dental Physician (vs. General Nurse)	0.169	0.180	-0.185, 0.523	0.94	0.349
Dental Nurse (vs. General Nurse)	-0.100	0.279	-0.648, 0.449	-0.36	0.721
Sex: Female (vs. Male)	-0.055	0.224	-0.496, 0.385	-0.25	0.805
Age category (per 1-step, 1–9 ordinal)	-0.008	0.027	-0.060, 0.045	-0.28	0.777
Years of experience (per 1-step, 0–7 ordinal)	0.015	0.022	-0.027, 0.058	0.71	0.478
Domicile: Urban (vs. Rural)	-0.144	0.102	-0.344, 0.055	-1.42	0.156
Prior training: Any (vs. None)	-0.076	0.163	-0.395, 0.243	-0.47	0.640

Notes. B, unstandardised regression coefficient; SE, conventional (ordinary least squares) standard error; CI, confidence interval. The dependent variable is the overall mean training-need score, computed as the average of the five domain means administered to all professional roles (communication; social and child rights; ethics; digital skills; common public-health items; 17 items in total). Reference categories: profession = General Nurse; sex = Male; domicile = Rural; prior training = None. Age category is an ordinal variable coded 1 (25–29 years) to 9 (over 65 years); years of experience was collected as a free-text numeric value and categorised into eight five-year bands coded 0 (0–4 years) to 7 (35 or more years). Model fit: n analysed listwise = 680; adjusted $R^2 = 0.005$; $F(8, 671) = 1.44$; overall $p = 0.177$. The dental physician contrast is exploratory given the small subgroup (n = 28). In a sensitivity analysis with heteroscedasticity-consistent (HC3) standard errors, the joint Wald test ($p = 0.118$) and the dental-physician contrast ($p = 0.299$) agreed with the conventional model (Section 2.7 of the manuscript).

Table S2.3. Sensitivity analysis: reliability and mean scores of the five domains administered to all professional roles, in the full consented sample (Main, N = 683) versus the generalist sub-sample with the dental subgroup excluded (Sensitivity, n = 644).

Domain	k items	Main n	Main mean (SD)	Main α (95% CI)	Sens. n	Sens. mean (SD)	Sens. α (95% CI)
Communication	4	683	3.55 (1.12)	0.951 (0.941, 0.959)	644	3.55 (1.13)	0.954 (0.945, 0.962)
Social and Child Rights	2	683	3.44 (1.11)	0.901 (0.876, 0.923)	644	3.44 (1.12)	0.903 (0.877, 0.924)
Ethics	2	683	3.24 (1.27)	0.962 (0.951, 0.972)	644	3.23 (1.28)	0.965 (0.953, 0.975)
Digital Skills	5	683	3.20 (1.12)	0.911 (0.895, 0.924)	644	3.20 (1.12)	0.914 (0.899, 0.927)
Public Health (common items)	4	683	3.17 (1.06)	0.872 (0.851, 0.891)	644	3.16 (1.07)	0.877 (0.857, 0.895)

Notes. α, Cronbach's alpha with 95% bootstrap confidence interval (1,000 resamples). The Main sample is the full consented analytic sample (N = 683; complete-case n per domain shown). The Sensitivity sample (n = 644) excludes all 39 dental personnel and is restricted to generalist school physicians and nurses. Following the reclassification of the health-education items as role-specific, this comparison covers the five domains administered to all professional roles (17 items); the generalist-only domains (Health Education, Public Health General-HCP, Clinical General-HCP) are identical in both samples by construction. Across the five domains,

the absolute difference in domain mean did not exceed 0.012 and the absolute difference in Cronbach's α did not exceed 0.005; item-level mean differences across the 17 common items did not exceed 0.05 in absolute value, indicating that the dental subgroup does not materially influence the common-core reliability or mean estimates.

Exploratory factor analysis of the 17 items administered to all professional roles

The dimensionality of the 17 items administered to all professional roles was examined by exploratory factor analysis (principal axis factoring with direct oblimin rotation, $\delta = 0$) in IBM SPSS Statistics, on the consented analytic sample. The items were factorable (Kaiser–Meyer–Olkin measure = 0.917; all item-level KMO values > 0.85; Bartlett's test of sphericity $\chi^2(136) = 10{,}622.6$, $p < 0.001$). Horn's parallel analysis (95th percentile of 1,000 random-data eigenvalues) supported the retention of two factors: a broad general training-need factor and a distinct digital-skills factor (inter-factor r = 0.52), whereas the five-factor solution corresponding to the a priori domains, an exploratory and theoretically imposed solution, showed simple structure, with every item loading most strongly on its intended domain and 76.2% of the variance explained (extraction sums of squared loadings; 62.7% for the two-factor solution). Both solutions are reported in full below (Tables S2.4–S2.7). These analyses are sample-specific, post-hoc evidence of dimensionality; they do not constitute an a priori psychometric validation of the instrument.

Table S2.4. Initial eigenvalues and Horn's parallel-analysis thresholds for the 17 common items.

Component	Eigenvalue	Parallel-analysis 95th percentile	Retain
1	8.949	1.337	Yes
2	2.377	1.267	Yes
3	1.032	1.217	No
4	0.969	1.174	No
5	0.649	1.140	No
6	0.504	1.106	No
7	0.480	1.074	No
8	0.344	1.046	No
9	0.322	1.016	No
10	0.292	0.988	No
11	0.217	0.961	No
12	0.204	0.933	No
13	0.169	0.905	No
14	0.164	0.879	No
15	0.153	0.850	No
16	0.110	0.820	No
17	0.067	0.782	No

Notes. Parallel-analysis thresholds are the 95th percentiles of eigenvalues from 1,000 random datasets of identical dimensions. A component is retained when its eigenvalue exceeds the corresponding threshold; two factors met this criterion.

Table S2.5. Pattern matrix of the two-factor solution (principal axis factoring, direct oblimin).

Domain	Item	F1 General	F2 Digital	h^2
Communication	Communicating with children (age-appropriate)	0.89		0.72
	Communicating with parents	0.86		0.68
	Communicating with authorities (school, municipality, county public health directorate)	0.94		0.78

	Motivation and persuasion	0.86		0.73
Social and Child Rights	Rights of the child	0.57		0.51
	Child abuse (physical, neglect, emotional, sexual)	0.50		0.45
Ethics	Data confidentiality	0.79		0.65
	Informed consent for minor patients	0.79		0.66
Digital Skills	Basic office software (word processing and spreadsheets)		0.62	0.52
	Data entry in the national health insurance information system		0.90	0.75
	Electronic patient records		0.92	0.82
	Email communication		0.77	0.65
	Use of generative artificial intelligence assistants		0.81	0.64
Public Health (common items)	Legislation governing school medical offices	0.67		0.49
	Active surveillance of chronically ill children	0.77		0.59
	Medical waste management	0.79		0.58
	Sterilisation and disinfection of instruments and equipment	0.58		0.43

Notes. h^2, extraction communality. F1, broad general training-need factor; F2, digital-skills factor. Inter-factor correlation r = 0.52. Pattern coefficients from the rotated solution (oblimin with Kaiser normalization); loadings with absolute value below 0.30 are suppressed, per the output convention. Extraction sums of squared loadings: 62.7% of variance (cumulative).

Table S2.6. Pattern matrix of the five-factor solution corresponding to the a priori domains (exploratory, theoretically imposed).

Domain	Item	F1	F2	F3	F4	F5	h^2
Communication	Communicating with children (age-appropriate)	0.88					0.85
	Communicating with parents	0.95					0.87
	Communicating with authorities (school, municipality, county public health directorate)	0.79					0.85
	Motivation and persuasion	0.73					0.78
Social and Child Rights	Rights of the child			0.86			0.85
	Child abuse (physical, neglect, emotional, sexual)			0.88			0.80
Ethics	Data confidentiality					0.92	0.91
	Informed consent for minor patients					0.94	0.94
Digital Skills	Basic office software (word processing and spreadsheets)		0.65				0.54
	Data entry in the national health insurance information system		0.89				0.76
	Electronic patient records		0.90				0.83
	Email communication		0.80				0.67
	Use of generative artificial intelligence assistants		0.76				0.64
Public Health (common items)	Legislation governing school medical offices				0.37		0.50
	Active surveillance of chronically ill children				0.66		0.69

	Medical waste management	0.97	0.89
	Sterilisation and disinfection of instruments and equipment	0.73	0.60

Notes. h^2, extraction communality. F1, Communication; F2, Digital Skills; F3, Social and Child Rights; F4, Public Health (common items); F5, Ethics — factor labels assigned from the highest-loading items. Every item loads most strongly on the factor corresponding to its a priori domain; 76.2% of variance explained (extraction sums of squared loadings). Pattern coefficients from the rotated solution (oblimin with Kaiser normalization); loadings with absolute value below 0.30 are suppressed, per the output convention; factors 3 and 5 were reflected (multiplied by −1) for readability, which does not affect the solution.

Table S2.7. Inter-factor correlations of the five-factor solution.

	F1	F2	F3	F4	F5
F1	1.00	0.39	0.53	0.66	0.66
F2	0.39	1.00	0.51	0.42	0.41
F3	0.53	0.51	1.00	0.52	0.63
F4	0.66	0.42	0.52	1.00	0.63
F5	0.66	0.41	0.63	0.63	1.00

Notes. Factor correlations from the direct-oblimin rotation of the five-factor solution (absolute values 0.39–0.66; factors 3 and 5 reflected, consistently with Table S2.6). For the two-factor solution, the correlation between the general and digital-skills factors was r = 0.52.
