# Supplementary material for: Self-Reported Training Needs and Continuing-Education Preferences of the School Medicine Workforce in Romania: A National Cross-Sectional Survey
Source: Healthcare (Basel). 2026 Oct 1;14(19):3245. doi: 10.3390/healthcare14193245 (PMC13664956; doi:10.3390/healthcare14193245)
Supplement: Supplementary file 1 [file healthcare-14-03245-s001.zip › 2026.09.24_healthcare-4558549_S3_R3.pdf]

Supplementary Material S3. Category definitions and example statements for the codebooks used in the content analysis of the two open-ended items.

Free-text responses to the two open-ended items were analysed by directed content analysis against written codebooks (Section 2.7 of the manuscript). Panel (a) documents the codebook for the additional-training-topics item; panel (b) documents the codebook for the barriers item. Example statements are verbatim respondent answers, with original spelling and diacritics retained and English translations in square brackets.

(a) Additional training topics — 53 specific codes consolidated into 13 mutually exclusive theme groups

Theme group	Specific codes consolidated	Example statements (verbatim)
Specific clinical conditions and procedures (12 specific codes)	Vaccination; ENT pathology / hearing / audiometry; administration of medicines; dermatological conditions; ultrasound; clinical guidelines; somatic medicine; pathological changes of the skeleton; child-specific pathologies / paediatrics; sample collection / injections / infusions / techniques; dental treatment in children with specific conditions; sterilisation	*"Vaccinologie"* [Vaccinology]<br>*"Sterilizarea"* [Sterilisation]
Legislation, rights, and professional roles and responsibilities (6 specific codes)	Legislation / rights / professional roles; school medical scholarships; clarification of the school physician's duties; malpractice; reporting to public-health authorities (DSP) and statistical reporting; employee–employer relations / office equipment and supply	*"Legislația în domeniul sănătății"* [Health legislation]<br>*"Drepturile pacientilor"* [Patients' rights]
Child and adolescent psychology and mental health (6 specific codes)	Neurodevelopmental disorders / autism / ADHD; child counselling; child neuropsychiatry; psychopedagogy; child and adolescent psychology; paediatric psychiatry	*"Psihologia copilului"* [Child psychology]<br>*"Psihiatrie pediatrica"* [Paediatric psychiatry]
Emergencies, first aid, and acute care (3 specific codes)	Emergency / first aid / urgent care; first aid combined with a specific clinical topic; trauma / surgery	*"Medicina de urgență"* [Emergency medicine]<br>*"Cursuri de prim.ajutor"* [First-aid courses]
Dietary investigation, menus, nutrition, and dietetics (5 specific codes)	Food allergies and intolerances; dietary investigation / menus / nutrition / dietetics; obesity; obesity (specific programmes); recipe collections for healthy dishes	*"Ancheta alimentara"* [Dietary investigation]<br>*"Nutritia copilului"* [Child nutrition]
Communication in specific professional contexts (1 specific code)	Communication in specific contexts (medical office–school management; pupils; parents; teaching staff)	*"Comunicarea cu elevii"* [Communication with pupils]<br>*"Comunicare cabinet medical - conducere unitatae scolara"* [Communication between the medical office and school management]
Collaboration with parents, colleagues, institutions, and other specialities (2 specific codes)	Collaboration (colleagues, parents, institutions, other specialities, the health system); leadership and teamwork	*"Colaborarea cu parintii si profesorii"* [Collaboration with parents and teachers]<br>*"Colaborare interdepartamentala"* [Interdepartmental collaboration]
Addictions, drug use, and risk behaviours (2 specific codes)	Screen dependence; addictions / drugs / risk behaviours	*"Consumul de droguri"* [Drug use]<br>*"Fumat, droguri"* [Smoking, drugs]

Theme group	Specific codes consolidated	Example statements (verbatim)
Digital skills and information technology (3 specific codes)	Effects of digital interaction; cybersecurity / data protection / artificial intelligence; computer use / IT	*"Utilizarea calculatorului"* [Computer use] *"Efectele relationării digitale"* [Effects of digital interaction]
Health education, including sexual education (1 specific code)	Health education (specific content, including sexual education and healthy lifestyle)	*"Educatia pentru sanatate"* [Health education] *"Educație sexuala pe înțelesul elevilor cu vârsta între 6 și 15 ani"* [Sex education adapted to the understanding of pupils aged 6 to 15]
Children with special educational needs (1 specific code)	Children with special educational needs (CES)	*"Copii cu CES"* [Children with special educational needs]
Other specific topics (9 specific codes)	Practical skills (general); adult-trainer certification; preparing and presenting a conference paper; workplace stress management; school medical office management; time management for pupils; the systemic role of epidemiological triage; natural therapies; other specific mentioned topic	*"Parodontologie"* [Periodontology] *"Terapii naturiste"* [Natural therapies]
No valid suggestion (2 specific codes)	No additional domains needed / current list sufficient; vague or uninterpretable response	*"Nu este cazul"* [Not applicable] *"Nu, multumesc"* [No, thank you]

Note. Each response was assigned one code from a framework of 53 specific codes, consolidated into the 13 mutually exclusive theme groups above; each response counted once, under its dominant theme group. Specific-code labels are English renderings of the category labels in the study dataset; theme-group labels correspond to Section 3.6 of the manuscript.

(b) Barriers to participation in continuing-education activities — nine coding categories and one residual group (no substantive answer)

Category	Definition	Example statements (verbatim)
Time / schedule constraints	Lack of time; busy/overloaded schedule; event overlaps the respondent's work programme or school year; time outside working hours; weekends consumed.	*"Lipsa de timp în afara programului de lucru"* [Lack of time outside working hours] *"Se suprapune cu orarul de lucru"* [It overlaps with the work schedule]
Cost	Any mention of money: course fees, travel/accommodation expense, lack of funds, taxes, lack of reimbursement/sponsorship, "free courses missing".	*"Costurile ridicate"* [High costs] *"Costuri suplimentare"* [Additional costs]
Limited local availability of training events	Absence or scarcity of organised events (in region/county/city/for own professional group); events only far away / hard-to-reach location; distance or travel difficulty; limited places.	*"Absenta in regiune"* [Absence [of events] in the region] *"Lipsa oportunitati"* [Lack of opportunities]
Relevance or adaptation of available content	Topics not adapted to needs / scope of school medicine; uninteresting, repetitive, outdated or non-specific themes.	*"Teme neadaptate nevoilor"* [Topics not adapted to needs] *"Teme neadecvate"* [Unsuitable topics]
Organisational or employer issues	Employer/workplace obstacles: no replacement staff, not released from work, employer refuses to pay or grant leave, must use own leave days, imposed courses, deficient legal/contract provisions.	*"Nu putem lăsa locul de munca ,nu are cine să înlocuiască."* [We cannot leave the workplace; there is no one to cover] *"Faptul că nu are cine să mă înlocuiască la serviciu când sunt organizate cursurile"* [The fact that there is no one to replace me at work when courses are organised]

Category	Definition	Example statements (verbatim)
Information or communication gaps	Lack of information / announcement about events; not knowing about events, their themes, place or timing.	*"Nu suntem anunțați de aceste cursuri"* [We are not informed about these courses]<br>*"Absenta cursurilor si neanuntarea lor in cazul in care exista"* [The absence of courses, and their not being announced when they do exist] (also coded: Limited local availability of training events)
Format of training events	How events are organised/delivered: missing online or physical option, weekend-only event scheduling, poorly organised, lack of practical/hands-on format, online credit-mill format.	*"costuri mari, absenta variantei online, cursuri sambata,"* [high costs, the absence of an online option, courses on Saturdays] (also coded: Cost)<br>*"Sunt prea putine in primul rand iar in al doilea rand prost organizate"* [Firstly there are too few, and secondly they are poorly organised] (also coded: Limited local availability of training events)
Specific personal circumstances	Personal circumstances: own health, family care obligations, personal qualification limits, additional personal work commitments.	*"Imposibilitatea deplasării la Congrese din motive familiale"* [Being unable to travel to congresses for family reasons]<br>*"Lipsa timp uneori, probleme de sănătate..."* [Sometimes lack of time, health problems…] (also coded: Time / schedule constraints)
No barriers reported	Explicit statement of no barriers, "not applicable", or a bare "no".	*"Nu am"* [I have none]<br>*"Niciuna"* [None]
No substantive answer	Placeholder character only ("-" or "/"), "do not know", or an off-topic remark; the item was compulsory in the deployed form, so a placeholder was the only way to leave it unanswered.	*"-"* [placeholder character]<br>*"nu știu"* [I do not know]

Note. Coding was multi-label: a response could endorse any number of the eight barrier categories, while "No barriers reported" and the residual group "No substantive answer" (a placeholder character entered to pass the compulsory item, "do not know", or an off-topic remark; 14 responses) are each mutually exclusive with all other categories. Generic responses of the type "Toate cele de mai sus" [All of the above] were coded to the four barriers given as examples in the questionnaire item (high costs, lack of time, topics not matching needs, and lack of locally organised events). Category definitions are reproduced verbatim from the coding workbook. Where a quoted example was additionally coded to other categories, these are given in parentheses.
